# Temperature increase modifies susceptibility to Verticillium wilt in *Medicago spp* and may contribute to the emergence of more aggressive pathogenic strains

**DOI:** 10.3389/fpls.2023.1109154

**Published:** 2023-02-14

**Authors:** Abed Al Latif Sbeiti, Mélanie Mazurier, Cécile Ben, Martina Rickauer, Laurent Gentzbittel

**Affiliations:** ^1^ Laboratoire d’Ecologie Fonctionnelle et Environnement, Université de Toulouse, CNRS, INPT, UPS, Castanet-Tolosan, France; ^2^ Project Center for Agro Technologies, Skolkovo Institute of Science and Technology, Moscow, Russia

**Keywords:** experimental evolution, mutation, temperature-adapted pathogens, global warming, virulence, aggressiveness, plant disease

## Abstract

Global warming is expected to have a direct impact on plant disease patterns in agro-eco-systems. However, few analyses report the effect of moderate temperature increase on disease severity due to soil-borne pathogens. For legumes, modifications of root plant-microbe interactions either mutualistic or pathogenic due to climate change may have dramatic effects. We investigated the effect of increasing temperature on the quantitative disease resistance to *Verticillium* spp., a major soil-borne fungal pathogen, in the model legume *Medicago truncatula* and the crop *M. sativa*. First, twelve pathogenic strains isolated from various geographical origin were characterized with regard to their *in vitro* growth and pathogenicity at 20°C, 25°C and 28°C. Most of them exhibited 25°C as the optimum temperature for *in vitro* parameters, and between 20°C and 25°C for pathogenicity. Second, a *V. alfalfae* strain was adapted to the higher temperature by experimental evolution, *i.e*. three rounds of UV mutagenesis and selection for pathogenicity at 28°C on a susceptible *M. truncatula* genotype. Inoculation of monospore isolates of these mutants on resistant and susceptible *M. truncatula* accessions revealed that at 28°C they were all more aggressive than the wild type strain, and that some had acquired the ability to cause disease on resistant genotype. Third, one mutant strain was selected for further studies of the effect of temperature increase on the response of *M. truncatula* and *M. sativa* (cultivated alfalfa). The response of seven contrasted *M. truncatula* genotypes and three alfalfa varieties to root inoculation was followed using disease severity and plant colonization, at 20°C, 25°C and 28°C. With increasing temperature, some lines switched from resistant (no symptoms, no fungus in the tissues) to tolerant (no symptoms but fungal growth into the tissues) phenotypes, or from partially resistant to susceptible. Further studies in greenhouse evidence the reduction in plant fitness due to disease in susceptible lines. We thus report that root pathogenic interactions are affected by anticipated global warming, with trends towards increased plant susceptibility and larger virulence for hot-adapted strains. New threats due to hot-adapted strains of soil-borne pathogens, with possibly wider host range and increased aggressiveness, might occur.

## 1 Introduction

Global climate change will affect a wide range of human and natural systems. The phenomenon and its impact will continue and even grow for many decades (IPCC 2014). One of its numerous effects is that distribution and abundance patterns of plants and their pathogens will probably change (Bebber, 2015), albeit at different rates. It is generally acknowledged that climate change may influence pathogenic interactions *via* three major effects. It may i) alter the biology of the pathogen and/or change the population structure of the pathogen, ii) alter the development and/or the metabolism of the plant, thus affecting all stages of disease development, and iii) lead to the emergence of a “new” disease, by increasing the geographic area of a pathogen, enlarging its host range, or by modifying disease severity (Anderson et al., 2004; Garrett et al., 2006; Bebber et al., 2014). Modifications of the interactions between host, pathogen and/or climate may result in an increase in disease severity or incidence, but may also have the opposite effect (Chakraborty, 2013). Climate changes will influence soil pathogens both directly (warming and soil moisture modifications) and indirectly *via* changes in quantity and quality of plant-mediated soil C inputs and modifications of nutritional quality of the plants (warming and elevated CO2) (McElrone et al., 2005; Stiling and Cornelissen, 2007; Pritchard, 2011).

The soil-borne fungal pathogens of the genus *Verticillium* have over 400 host species (Pegg and Brady, 2002), from annual herbs (*Arabidopsis*, *Medicago*) to trees (maple, olive tree) and vegetables (tomato). *Verticillium* spp. colonizes the vascular tissue of roots and then of shoots, causes typical wilt symptoms and leads to the death of susceptible plants. The *V. albo-atrum* is usually present in cooler regions than *V. dahliae* (Pegg and Brady, 2002). The former *V. albo-atrum* species was further split into three species, i.e. *V. albo-atrum sensu stricto*, *V. alfalfae* and *V. nonalfalfae* (Inderbitzin et al., 2011; Inderbitzin et al., 2013; Inderbitzin and Subbarao, 2014). Hence, it is not possible to relate the previous literature on “*V. albo-atrum*” with absolute certainty to the current species concepts of *V. albo-atrum sensu stricto*, *V. alfalfae* and *V. nonalfalfae*. Verticillium wilt of alfalfa (*Medicago sativa*) due to *V. alfalfae* (previously *V. albo-atrum* ‘alfalfa’ strains) or due to *V. nonalfalfae* (Inderbitzin and Subbarao, 2014) has been reported in very diverse conditions, from cold-temperate European climate (Richter and Klinkowski, 1938) to South-Californian desert (Erwin et al., 1988). In North-America, the disease was first reported in 1962 in Quebec, in 1973 in Washington State (Acharya and Huang, 2003) and in 1988 in Southern California, where it can occur at mean temperatures greater than 35°C (Howell and Erwin, 1995; Erwin and Howell, 1998). This suggests that *Verticillium* spp. already adapted rapidly to warmer temperature, and may respond quickly to temperature increases that are anticipated during climate change. Heale (1985) reported that *V. albo-atrum* alfalfa strains (i.e. *V. alfalfae* under the new taxonomy) have a higher optimum temperature than strains from other hosts.

Since legume plants establish symbiotic interactions in their roots with nitrogen-fixing rhizobia (Oldroyd, 2013), they are of utmost importance for sustainable agriculture and ecosystems, and are a valuable source of protein for food and feed even in poor soil conditions. *Medicago truncatula* (barrel medic) is a wild annual legume species that occurs naturally around the Mediterranean basin and is adapted to a variety of different soils and climates. It has high synteny with cultivated grain legume crops such as pea, and is closely related to cultivated alfalfa (Li et al., 2014; Chen et al., 2020). *M. truncatula* has been adopted as a model for legume (see for review Gentzbittel et al., 2015; Garmier et al., 2017). Climate models predict that strong modifications will occur in the Mediterranean Basin, particularly during the winter and spring months, which correspond to the growth and reproduction periods of *M. truncatula*. Hence *M. truncatula* is an excellent model for studies aiming to understand plant adaptation to pathogens in a context of climate change. Similarly to alfalfa, *M. truncatula* is a host for *Verticillium* spp. (Mace, 2012; Ben et al., 2013; Negahi et al., 2013). Resistance to *Verticillium* spp. is often considered as quantitative disease resistance (QDR) in numerous plants such as *Arabidopsis thaliana*, alfalfa and woody species (Mace, 2012). QDR is characterized by a continuous range of phenotypes within segregating or natural populations (Poland et al., 2009). It is conditioned by multiple genes of often small effects, which may further interact with the environment (Roux et al., 2014). QDR may lead to a total absence of symptoms in genotypes gathering all favorable alleles for resistance. QDR is not specific of pathogen race offering thus a broader spectrum, and is presumably more durable due to its polygenic inheritance (Roumen, 1994; Xu et al., 2012). In alfalfa, two tightly linked resistance and susceptibility genes were recently identified in the same ecotype, highlighting the complex response to Verticillium wilt in legumes (Lin et al., 2022). It is generally considered that disease is favoured by temperatures which are optimal for the pathogen but less so for the host plant (Agrios, 2005). In the case of Verticillium wilt of olive tree (Calderón et al., 2014) it has been shown that soil temperature influenced disease development, with genotype effects for both pathogen strain and host plant, and studies in cotton described the effect of temperature on fungal growth parameters and disease symptoms (Xu et al., 2012). Genetic analyses performed on crosses of various *M. truncatula* genotypes showed that Verticillium wilt response in that species is a QDR, regulated by QTL that differ across resistant accessions and according to the fungus strains (Ben et al., 2013; Negahi et al., 2014). Resistance in *M. truncatula* Jemalong A17 is associated with transcriptional responses related to innate immunity (Toueni et al., 2016). It has been also reported that cross-talk between symbiotic and defense signaling pathways exist, as several *M. truncatula* mutants affected in nodulation are also affected in their response to fungal pathogens (Ben et al., 2013; Rey et al., 2013; Rey et al., 2016). Hence, modifications of plant-microbe interactions due to climate change may have dramatic effects in legumes by modifying symbiotic and pathogenic relationships in natural and agro-systems.

Without additional mitigation of greenhouse gases emissions, increases in 2100 from 3.7°C to 4.8°C (median values; the range is 2.5°C to 7.8°C when including climate uncertainty) compared to pre‐industrial levels are predicted (IPCC, Masson-Delmotte et al., 2021). In this study we thus tested if moderate increases in temperature (+3°C to +5°C) would modify net patterns of partial resistance to a soil-borne pathogen. As a first step in evaluating the effect of global warming on QDR, the net effects of temperature increase on root infection at early stage of plant development were studied. The interaction of *M. truncatula* and *M. sativa* with *V. alfalfae* strains that are adapted to moderate (25°C) and warm (28°C) temperatures, respectively, were then investigated at three temperatures regimes (20/20°C, 25/23°C, and 28/26°C). For both legume species, disease severity and survival rate was assessed during four weeks after fungal infection. The effect of infection at different temperature on global fitness traits of *M. truncatula* was assessed in green house at the end of the plant’s cycle.

## 2 Materials and methods

### 2.1 Plant material

Seven *M. truncatula* lines derived from natural accessions of distinct geographic origins around the Mediterranean basin (**Supplementary Table 1A**) and three varieties of *M. sativa* adapted for the Mediterranean region (**Supplementary Table 1B**) were used. These plant genotypes have been chosen also for their previously reported contrasted response towards *V. alfalfae* strain V31-2 (Molinéro-Demilly et al., 2007; Ben et al., 2013; Negahi et al., 2013). Seed germination, and plant culture in peat substrate (Jiffy-7, Jiffy International AS, Norway) were performed as described by Ben et al. (2013). The growth chamber conditions were 16 h of light (at 170 μmol m^–2^ s^–1^) at 25°C and 8 h of darkness at 23°C for one week before inoculation. Plants were inoculated with *Sinorhizobium meliloti* (strain RCR2011) two days after germination, by adding 200µl of a suspension at 10^7^ Colony Forming Unit.mL^-1^ at the basis of the stem.

### 2.2 Fungal isolates

Twelve *Verticillium* strains from various host plants and geographic origins sampling different temperature zones, were used (**Supplementary Table 2**), and were grown on Potato Dextrose Agar (PDA) medium at 24°C in the dark.

### 2.3 *Verticillium* mutagenesis and selection of isolates adapted to warm temperatures

We selected hot-adapted strains using UV mutagenesis of the *V. alfalfae* V31-2 strain (Buxton and Hastie, 1962). Conidia were exposed to UV radiation (254 nm) during 12 seconds (LD50 previously determined, **Supplementary Figure S1**), resulting mycelium was grown at 24°C for two weeks and the newly formed conidia were used to inoculate the susceptible *M. truncatula* line F83005.5 at 28°C. The inoculated plants were maintained at 28°C and the symptoms were scored during 4 weeks. The disease severity was rated using a scale from 0 (no symptoms) to 4 (dead plants), as described in **Figure 1** of Ben et al. (2013). The fungus was re-isolated from aerial parts of plants presenting symptom scores of ≥ 2. Re-isolated mycelium was incubated at 24°C to produce conidia, which were again submitted to a round of mutagenesis, conidia production, plant inoculation and re-isolation. This mutagenesis/selection/re-isolation cycle was repeated three times to increase selection intensity. After the third round, 180 randomly selected mono-conidium strains were isolated. Among these, strain AS38, which presented good growth, sporulation capacity and pathogenicity at 28°C was retained as a “warm temperature-adapted” strain.

**Figure 1:**
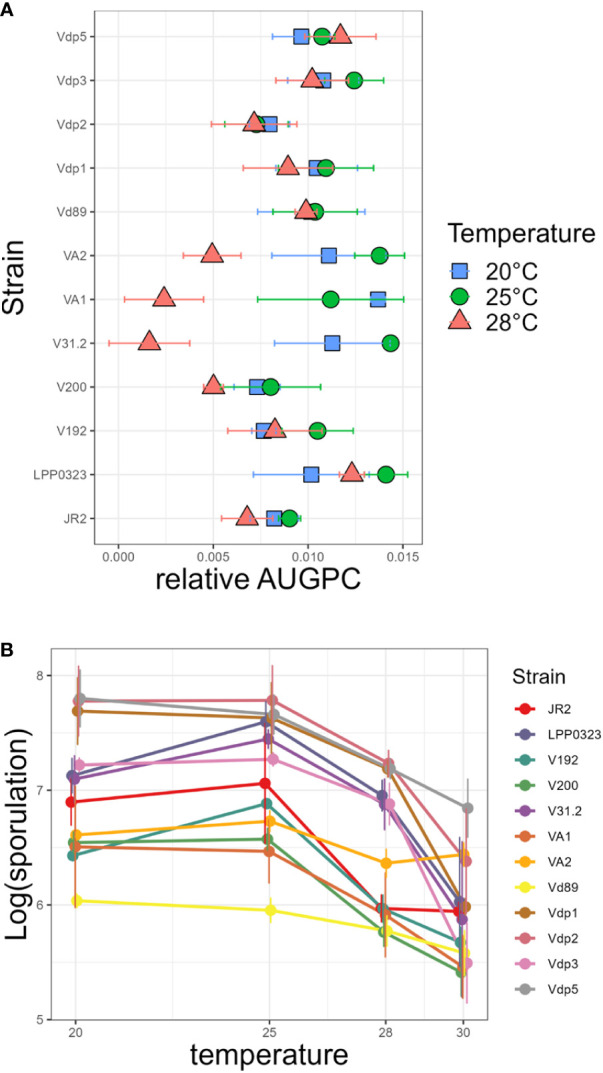
Effect of temperature on *in vitro* growth and sporulation of *Verticillium* strains. **(A)** strains were grown on PDA medium in Petri dishes at 20°C, 25°C and 28°C. Radial growth was measured during 14 days and expressed as Area Under Growth Progress Curve (AUGPC). **(B)** Conidia released into distilled water were counted with a hematocytometer. Each point shows the mean values of three independent experiments, with two Petri dishes each. Bars indicate standard error of observed values.

### 2.4 Inoculation and symptom scoring

Ten day-old plants were inoculated with a fungal spore suspension at a concentration of 10^6^ spores per ml, as described by Ben et al. (2013). *Verticillium*- and mock-inoculated plants were transferred into growth chambers with a photoperiod of 16 h and three different day/night regimes: 20°C/20°C, 25°C/23°C and 28°C/26°C. Individual plants’ symptoms were scored regularly during four weeks, and disease severity was rated as described in Ben et al. (2013). Eight plants per *M. truncatula* line were used for each condition (combinations of temperature regime and pathogen strains), and 100 plants for each *M. sativa* variety (only scoring dead plants). Three independent replicates of the whole design were done. We considered plants as fully resistant when pathogen growth in the xylem was very limited and few, if any, symptoms occurred. Susceptibility is defined as the state in which the fungus proliferates systemically throughout the plant leading to symptom expression and disease development. Partial resistance represents intermediate symptom expression. A plant is disease tolerant if extensive *Verticillium* colonization occurs systemically, but few, if any, symptoms are expressed (Beckman and Roberts, 1995; Gao et al., 1995; Roy and Kirchner, 2000).

### 2.5 Fungal growth measurement and sporulation performance

Plugs of mycelium (diameter 5mm) from the periphery of growing cultures were placed on fresh PDA plates and the Petri dishes were incubated in the dark at 20°C, 25°C, 28°C and 30°C. Two Petri dishes were made for each fungal strain and condition, and three independent experiments were performed. Radial growth was measured on two perpendicular radii for each culture, every second day during two weeks, and the mean values for radius were calculated. Spore suspensions were obtained after two weeks of culture by flooding the Petri dishes with 20 ml of sterile water. Their concentration was determined with a hemocytometer.

### 2.6 *Verticillium* quantification *in planta* by q-PCR

Ten days after inoculation (i.e. onset of wilting symptoms in the most susceptible *M. truncatula* lines), the first unifoliate leaf was harvested and DNA was extracted as described by Ghérardi et al. (1998). *Verticillium* DNA was amplified using vert853F and vert927R primers described by Larsen et al. (2007), and *M. truncatula* DNA was amplified with actin primers (Ariel et al., 2010). Reactions were carried out in two replicates per sample in a 384-well plate containing 5µl of KAPA SYBR Fast ABI Prism Readymix Kit (Clinisciences, France), 2 µl primers (0.35µM) and using respectively 50ng/µl and 150ng/µl of DNA matrix for *Medicago* and *Verticillium* amplifications in 10 μl total reaction volume. Relative fungal biomass was estimated as the ratio of fungal DNA per unit of plant DNA, each determined by their cycle threshold, Ct (ΔCt = Ct *Verticillium* DNA – Ct *M. truncatula* DNA). Pools of eight plants were analysed in that way per *M. truncatula* x temperature condition, in two independent experiments.

### 2.7 *Verticillium* re-isolation from stem tissues

Thirty days after inoculation, 2-cm-long fragments of the main stem was cut above the first node, and was successively surface-sterilized in 70% ethanol for 15 seconds and in 0.96% commercial bleach for 6 minutes then rinsed thrice in sterile water for 1 minute. The stem sections were then incubated on PDA medium containing 50mg/ml streptomycin during three days at 25°C. The number of stem ends showing outgrowth of white mycelium was determined and percentage of re-isolation was calculated. This experiment was repeated three times independently. Presence of the fungus in reproductive parts (podes and seeds) was checked by disinfecting whole pods and seeds in the same way. Seeds were scarified with a sterile scalpel. Emptied pods and seeds were incubated on medium as described above.

### 2.8 Components of plant fitness in greenhouse

After four weeks of symptom scoring in phytotron at 20°C, 25°C and 28°C, six plants (three mock-inoculated and three *Verticillium*-inoculated) were chosen randomly for each *M. truncatula* line and each temperature, and transferred into the greenhouse for assessment of fitness in response to strain V31-2. Each plant was potted in soil-sand (2:1) mixture in 2L pots. This experiment was repeated twice in two following years, with three blocks per replicate. After two months of growth, each plant was wrapped in a net in order to recover all pods of an individual plant. Plants were harvested six months after transfer into the greenhouse. Number of pods, pod weight and aerial dry biomass per plant, as well as germination capacity of seeds were measured to assess plant fitness.

### 2.9 Statistical analysis

Area Under the Disease Progress Curve and Area Under the Growth Progress Curve (AUDPC and AUGPC, respectively, Shaner and Finney, 1977) were computed based on the disease scores and fungal radial growth respectively, using the ‘agricolae’ R package (De Mendiburu and Muhammad Yaseen, 2020; R Core team, 2022). For sporulation, data were analyzed using generalized linear model for counts data using Poisson regression (glm function of R). For *Verticillium* mutagenesis, LD50 was determined after fitting a generalized linear model for counts data as a function of time of exposition to UV light, with a binomial link function (glm function of R). For disease symptom scores (disease severity), data were analyzed using proportional-odds models (McCullagh, 1980; Agresti, 2012), using the orm function of the ‘rms’ R package (Harrell, 2015). The aim is to relate the dependence of the ordinal response (disease symptom scores) on discrete (pathogen strains and plant lines) or continuous (temperature) covariates. Effects of covariates were tested using analysis of deviance. Multiple comparisons of odd-ratio for symptom scores were done using least square means and Bonferonni correction using the ‘lsmeans’ R package (Lenth, 2016) after fitting a proportional-odds model for each temperature level. For disease incidence in *M. sativa* (percentage of dead plants at 28 dpi), data were analyzed using a generalized linear model for proportions using the glm function with the logit link. Fitness experiment in common garden was analyzed with ANOVA in a Randomized Complete Block Design model. When required, data transformations were applied to achieve normality and homoscedasticity of ANOVA residuals. Pairwise treatment differences were determined by a Tukey’s test using package ‘lsmeans’ or Newman-Keuls test using the ‘agricolae’ package. Graphs were drawn using the ‘ggplot2’ package (Wickham, 2009).

## 3 Results

### 3.1 Temperature optimum for *Verticillium* wilt of *M. truncatula* is 20°C to 25°C but artificial evolution allows for a shift towards 28°C

*Verticillium* strains from different geographic origins and host plants were screened for their optimal temperature in order to identify strains adapted to cool, temperate and warm climate. *In vitro* growth parameters were analysed at 20, 25, and 28°C. Hyphal growth as calculated by AUGPC showed that the 12 strains differed in temperature optima and in their range of tolerance to high temperatures (**Figure 1A**). Statistical analysis demonstrates highly significant strain × temperature interactions with P<0.001 (**Supplementary Table 3**). The optimal temperature for hyphal growth was 25°C except for strain Vdp5 (potato isolate from Israel) which grew best at 28°C and strain VA1 (potato isolate from Canada) which grew best at 20°C. Sporulation of these strains, determined after two weeks of growth, was also affected by temperature (**Figure 1B** and **Supplementary Table 4**). The temperature optimum for sporulation was shown to be 25°C for eight strains and 20°C for the four remaining strains (namely Vdp1, Vdp5, Vd89 and VA1); none sporulated better at 28°C.

**Figure 2:**
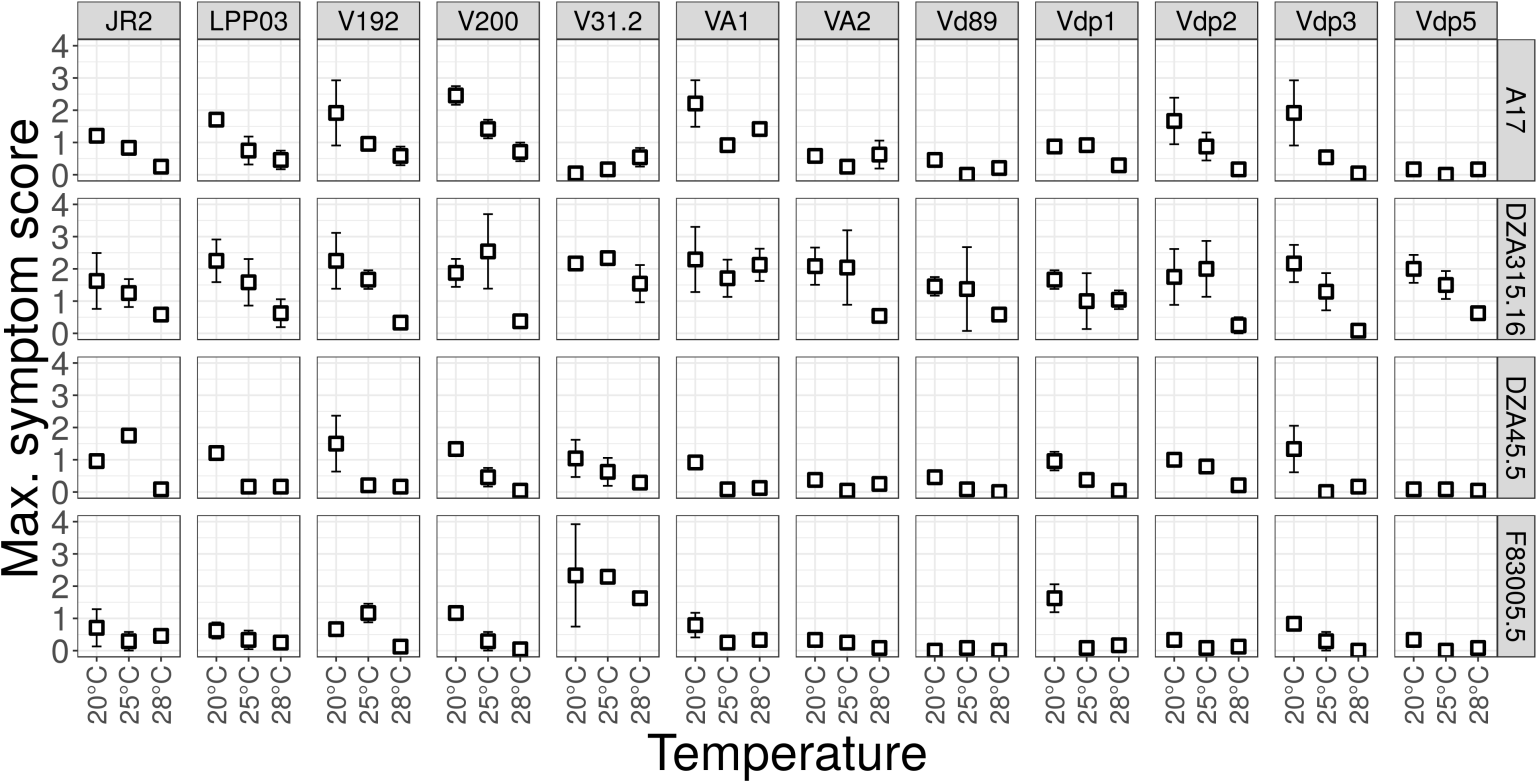
Aggressiveness of *Verticillium* strains on *M. truncatula* at different temperatures, shown by maximum symptom scores. Four *M. truncatula* lines (A17, DZA315.16, DZA45.5 and F83005.5) were root-inoculated with a spore suspension of the 12 *Verticillium* strains, and maintained at 20°C, 25°C and 28°C for four weeks. Symptoms were scored regularly on a scale from 0 to 4 and Maximum Symptom Scores (MSS) and Area Under the Disease Progress Curve (AUDPC, not shown) were determined at the end of the experiment. The values are means from three independent experiments, with eight plants each. Bars indicate standard error of observed values.

To test for optimal temperature of disease development, these 12 *Verticillium* strains were inoculated on four *M. truncatula* lines (A17, DZA45.5, F83005.5 and DZA315.16, **Figure 2**), at different day-night temperature regimes. A17 and DZA45.5 were previously described as resistant, and F83005.5 and DZA315.16 as susceptible to V31-2 at 20°C (Ben et al., 2013). The results show that Verticillium isolates from other host plants, e.g. *V. dahliae* V200 from cotton and *V. non-alfalfae* VA1 from potato, are able to cause disease on *M. truncatula*. Statistical analysis of the disease score at the end of the experiment (herein called Maximum Symptom Score, MSS) using proportional-odds models reveals a highly significant line × strain × temperature interaction, indicating that plant’s response to the pathogen strain depends on the temperature. (P<0.0001, **Supplementary Table 5**). In susceptible genotypes, the optimum temperature for disease induction was 20°C or 25°C depending on the fungal strain. The aggressiveness of strain V31-2 on the susceptible *M. truncatula* lines, and that of most other strains was highest at 25°C and 20°C, and lowest at 28°C. Strain Vdp5 which was well adapted to high temperature for *in vitro* growth, was weakly pathogenic on *M. truncatula* susceptible accessions (**Supplementary Table 6**). Results obtained with the AUDPC parameter were similar and did not allow identifying a *Verticillium* strain adapted to higher temperature (data not shown). Based on temperature optima for the three parameters *in vitro* growth, sporulation capacity and aggressiveness, none of these 12 strains seemed to be truly adapted to higher temperatures, to be used for studies of plant responses in a scenario of climate change.

**Figure 3:**
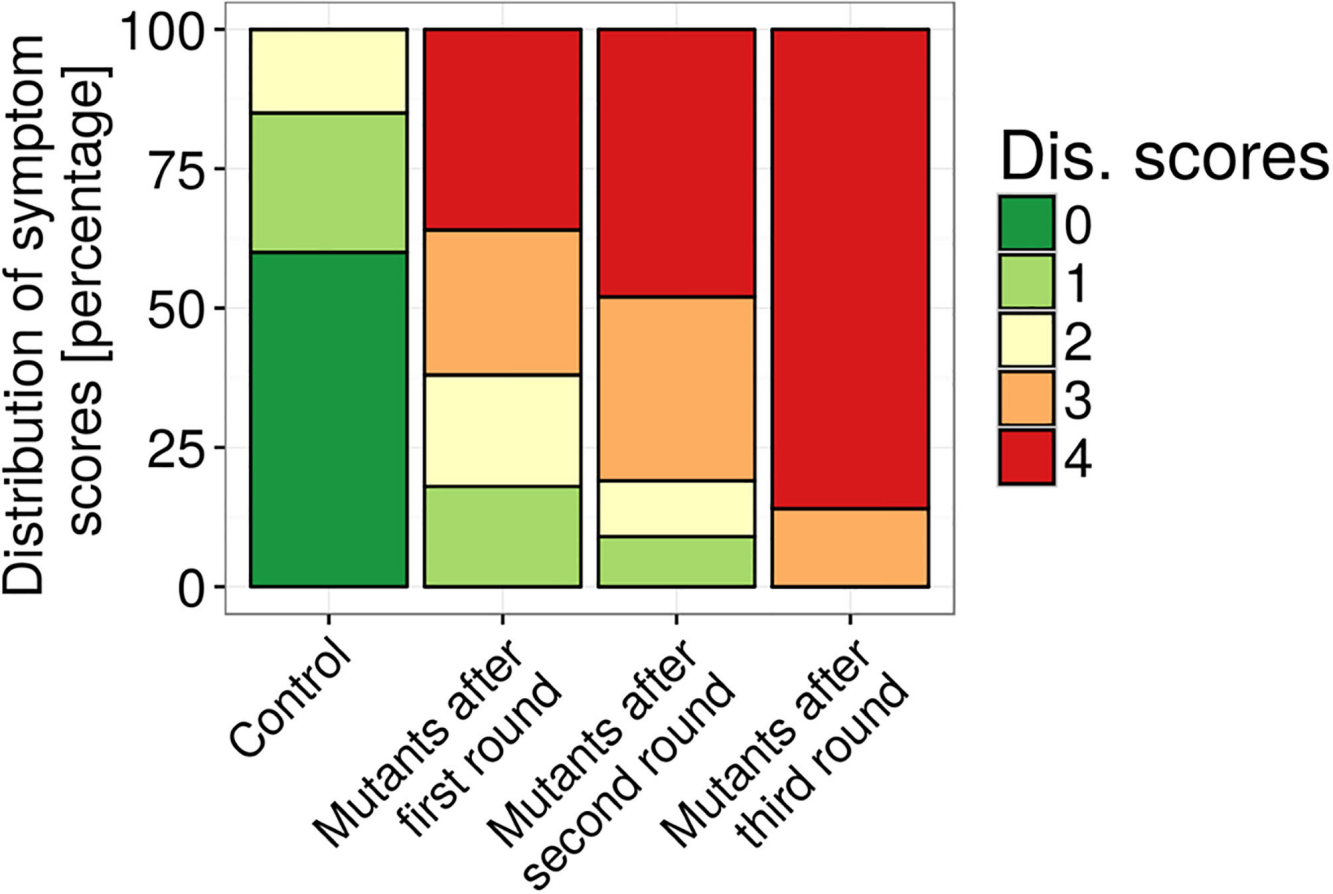
Aggressiveness of *V. alfalfae* V31-2 strains before and after successive steps of UV mutagenesis and *in planta* selection at 28°C. Plants of the susceptible line F83005.5 were root-inoculated and the symptom score after four weeks was determined, on a scale from 0 (healthy plant) to 4 (dead plant). One hundred fifty plants were inoculated for each cycle.

*V. alfalfae* (previously named *V. albo-atrum* ‘alfalfa strains’) has been reported to survive in the Mojave Desert (Erwin et al., 1988; Erwin and Howell, 1998) at temperature higher than 35°C. However, these strains are no longer available (A. Howell, personal communication). Hence we created a strain adapted to 28°C by successive cycles of UV mutagenesis, plant inoculation at 28°C and fungus re-isolation. As can be seen in **Figure 3**, the aggressiveness of mutated *V. alfalfae* increased with each cycle of inoculation/reisolation on the susceptible line F83005.5. At the end of this accelerated evolution, 86% of plants inoculated with the *V. alfalfae* mutants presented symptom scores of 4 (dead plants), whereas with the wild type strain the maximum score observed was 2 at 28°C and 60% of the plants were symptomless. Mono-conidium isolates were obtained from mutants after 3 rounds of mutagenesis. One-hundred eighty of these mono-conidium isolates from the collection were inoculated on *M. truncatula* line A17 (resistant to V31-2) and F83005.5 (susceptible to V31-2) at 28°C. This preliminary experiment revealed that all isolates showed adaptation to high temperature (**Supplementary Figure S2**), and that some of them seemed to have acquired the ability to cause mild symptoms on line A17, such as AS11, AS38 or AS90. The mono-conidium isolate named AS38 which showed similar discrimination between the two genotypes as the wild type was retained for further studies. Despite its infectious adaptation to higher temperatures, optimal temperature for *in vitro* growth and sporulation of this mutant was still 25°C (**Supplementary Figure S3**).

### 3.2 Responses of contrasted *M. truncatula* lines to *V. alfalfae* V31-2 and its warm temperature-adapted mutant reveal significant increase in aggressiveness at higher temperature for the adapted mutant

To get a detailed evaluation of the impact of temperature increase on the plant-pathogen interaction, the two *V. alfalfae* strains (V31-2 and its derived mutant AS38) were inoculated at three temperature regimes (20/20°C, 25/23°C, 28/26°C) on a set of seven *M. truncatula* lines that have been observed to show contrasted behavior with strain V31-2 at 20°C (Ben et al., 2013; Negahi et al., 2013 and this study). Statistical analysis of MSS using proportional-odds model showed a clear effect of temperature on disease severity with significant interactions with strains and plant accessions (**Table 1**). This confirms that the interaction of the plants with the wild strain V31-2 and with its warm-temperature-adapted mutant AS38 depends on temperature.

**Table 1:** Analysis of deviance of proportional-odds model for Maximum Symptom Score (MSS).

Factor	Chi-Square	d.f.	P
Strain (factor + higher order factors)	89.66	9	<.0001
All Interactions with Strain	75.77	8	<.0001
Temperature (factor + higher order factors)	54.94	16	<.0001
All Interactions with Temperature	47.27	14	<.0001
Line (factor + higher order factors)	476.05	24	<.0001
All Interactions with Line	85.8	18	<.0001
Strain **×** Temperature (factor + higher order factors)	19.09	2	0.0001
Strain **×** Line (factor + higher order factors)	57.44	6	<.0001
Temperature **×** Line (factor + higher order factors)	29.58	12	0.0032
TOTAL	484.71	29	<.0001

Chi-Square: reduction in deviance due to the added factor; d.f., degree of freedom; P, probability of getting observed ChiSquared-values under the null hypothesis. Terms are added sequentially (first to last). Seven *M. truncatula* lines were infected by *V. alfalfae* strains V31-2 and mutant strain AS38, at 20°C, 25°C and 28°C.

**Figure 4:**
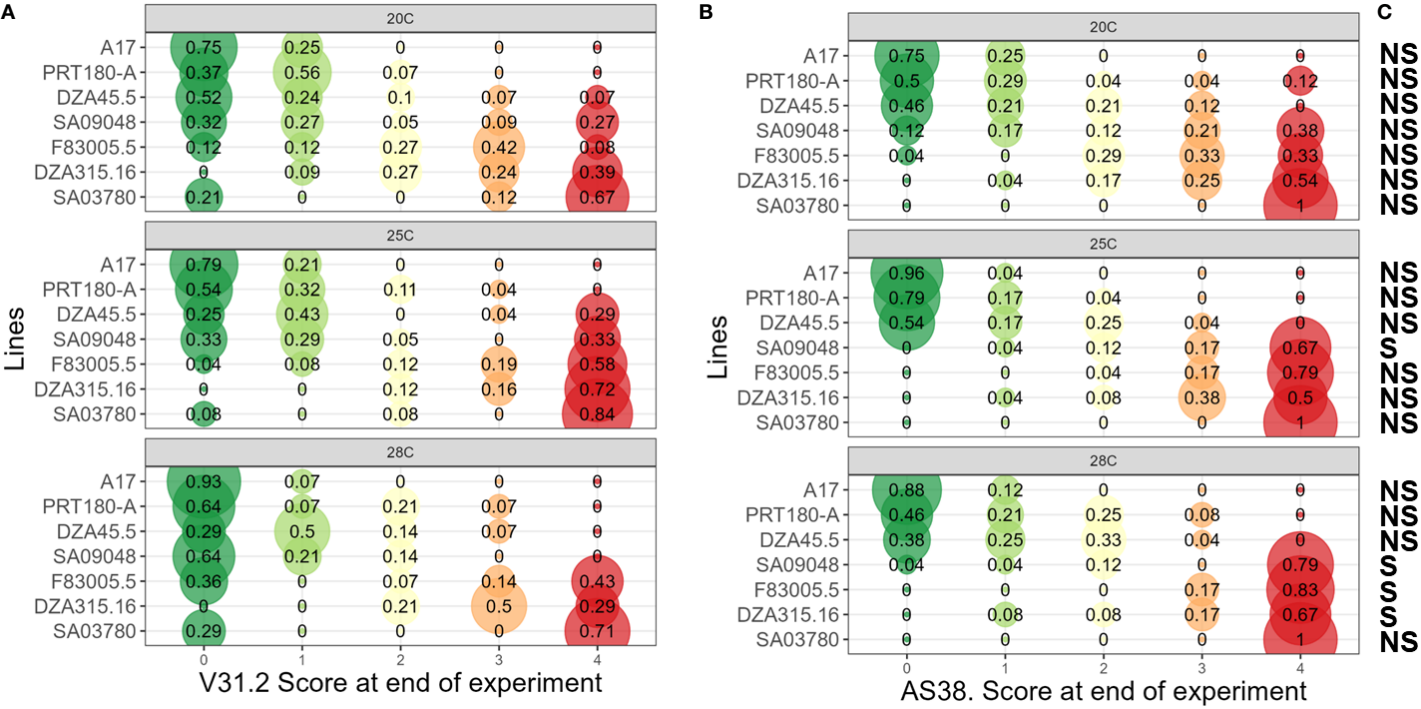
Assessment of Maximum Symptom Score of *M. truncatula* to *V. alfalfae* V31-2 and the AS38 derived mutant at different temperatures. Seven *M. truncatula* lines (A17, PRT180-A, DZA45.5, SA09048, F83005.5, DZA315.16 and SA03780) were root-inoculated with a spore suspension of Verticillium strains V31-2 **(A)** and AS38 **(B)** and maintained at 20°C, 25°C and 28°C. Symptoms were scored regularly on a scale from 0 to 4 during four weeks. At the end of the experiment, maximum symptom scores were determined for each plant. The values are from three independent experiments with at least eight plants per combination. Each bubble’s diameter is proportional to the percentage of plants belonging to a symptoms class, with this proportion indicated at the center. Colour code from green (score “0”, no symptoms) to red (score “4”, dead plant). Score classes at the end of the experiments were analyzed using proportional-odds models, used for modeling the dependence of an ordinal response (disease symptom scores) on discrete (pathogen strains and plant lines) or continuous (temperature) covariates. Multiple comparisons of odd-ratio were done using least square means and Tukey HSD. **(C)** NS/S indicates if a significant difference exists between the response of a *M. truncatula* accession to AS38 compared to its response to V31-2, for a given temperature.

Strain V31-2, considered to be adapted to a temperate climate, was most aggressive at 25°C, with MSS ≥ 3 on the three susceptible lines F83005.5, DZA315.16 and SA3780 (**Figure 4A**); but at 28°C these values decreased. The remaining lines A17, DZA45.5, and PRT180-A were resistant and the line SA9048 was partially resistant. The mutant strain AS38 which has been adapted to high temperatures was more aggressive than V31-2, notably at 28°C, and induced symptoms in the three lines susceptible to strain V31-2 (DZA315.16, F83005.5 and SA3780) and in addition in line SA9048, with MSS >3.5; the remaining three lines A17, DZA45.5 and PRT180-A were resistant (**Figure 4B**). At 20°C, the disease severity of V31-2 and AS38 is not significantly different (**Figure 4C**). At 25°C, the line SA9048 was significantly more affected with AS38 compared to V31-2. At 28°C, three lines exhibited a pattern of increased disease severity: SA9048, F83005.5, DZA315.16 (**Figure 4C** and **Supplementary Table 7**). In all cases, the line SA3780 is severely affected, with non-significant but noticeable increase of MSS when infected by AS38. MSS and AUDPC were positively correlated and AUDPC analysis revealed a similar pattern of variation (data not shown).

In order to evaluate if plant colonization capacity was modified in mutant AS38 compared to its wild type, and the putative influence of temperature, colonization of aerial parts by the fungus was assessed by microbiological re-isolation, and by quantification of fungal DNA by qPCR, in four lines inoculated by strain V31-2, in two independent experiments. **Supplementary Figure S4** represents fungal colonization quantified by qPCR as ΔCt versus quantification by re-isolation rates. ΔCt values were significantly and negatively correlated with colonization estimated by re-isolation (r = 0.79, P<0.01), at the three temperatures, i.e. less fungal DNA was detected (high ΔCt) when tissue colonisation was low. qPCR analysis is a non-destructive test which at an early stage (10 dpi) allows predicting plant colonization. Yet, as re-isolation has both the advantage of revealing alive pathogen and being more practicable, we systematically performed fungal re-isolation to assess plant response and pathogen fitness for all combinations of line × strain × temperature (**Table 2**). Analysis of deviance indicates very significant effect of line × strain × temperature interaction on re-isolation percentage (**Supplementary Table 8**), and shows that in all cases (except A17), re-isolation percentage at 28°C is significantly greater with AS38 compared to V31-2 strain. This suggests an improved fitness of the hot-adapted AS38 mutant at 28°C.

**Table 2:** Colonization rate of seven *M. truncatula* accessions infected by *V. alfalfae*.

	V31-2			AS38		
	20°C	25°C	28°C	20°C	25°C	28°C
A17	2 ± 0.7	0 ± 0.0	0 ± 0.0	0 ± 0.0	0 ± 0.0	0 ± 0.0
PRT180-A	5.3 ± 0.6	5.3 ± 0.6	20.3 ± 0.6	9.3 ± 1.0	13 ± 0.8	35.7 ± 1.6
DZA45.5	12 ± 1.1	16.3 ± 1.4	7.3 ± 0.9	36 ± 0.8	18 ± 1.1	60 ± 0.8
SA9048	17 ± 1.1	23.3 ± 1.4	11.7 ± 1.0	56 ± 1.4	71 ± 0.7	87.7 ± 1.0
F83005.5	52.3 ± 1.6	63 ± 1.2	50 ± 1.1	86.3 ± 1.3	90 ± 0.4	100 ± 0.0
DZA315.16	57.3 ± 0.8	68.3 ± 0.8	46.3 ± 0.6	63 ± 1.0	81 ± 0.4	100 ± 0.0
SA3780	71.7 ± 1.2	75.3 ± 1.4	56 ± 1.1	100 ± 0.0	100 ± 0.0	100 ± 0.0

Accessions were infected with strains V31-2 and AS38, at 20°C, 25°C and 28°C. After one month, two-cm-long fragments of the main stem was cut above the first node, and incubated on PDA medium during three days at 25°C. The number of stem ends showing outgrowth of white mycelium was determined and percentage of re-isolation was calculated. Data from three independent replicates, with at least 6 plants per replicate. Standard error of average percentage is indicated.

**Figure 5:**
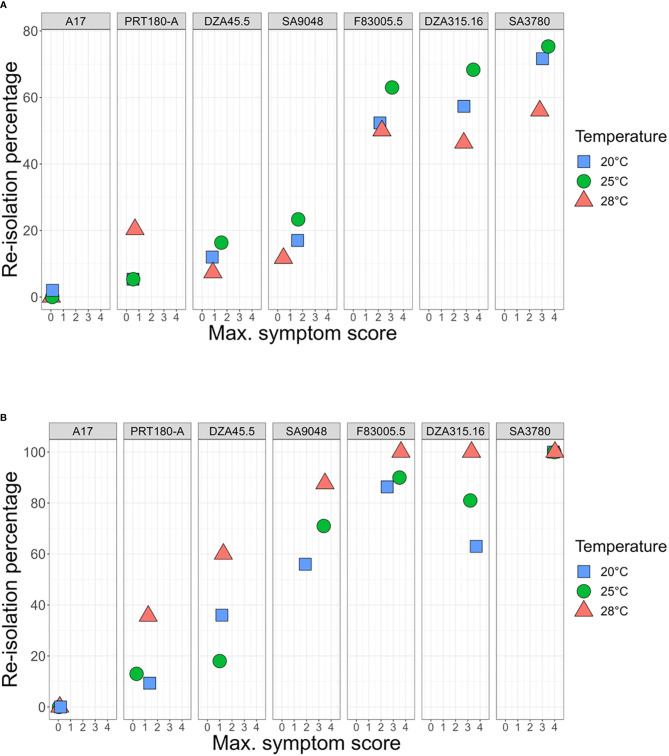
Assessment of Quantitative Disease Resistance (QDR) of *M. truncatula* to *V. alfalfae* V31-2 and AS38 its derived mutant at different temperatures, using Maximum Symptom Score and plant colonization evaluation. Seven *M. truncatula* lines (A17, PRT180-A, DZA45.5, SA09048, F83005.5, DZA315,16 and SA03780) were root-inoculated with a spore suspension of *Verticillium* strains V31-2 **(A)** and AS38 **(B)** and maintained at 20°C, 25°C and 28°C. Symptoms were scored regularly on a scale from 0 to 4 during four weeks. At the end of the experiment, maximum symptom scores were determined, and stem sections were harvested for fungus re-isolation. The values for MSS and re-isolation percentage are means from three independent experiments, with at least eight plants each. Bars indicate standard error of mean values.

Combining MSS data and plant colonization assessment allows a more detailed characterization of QDR at the different temperatures in response to the two pathogen strains. Joint representation of MSS data and re-isolation percentages of strain V31-2 from inoculated plants confirmed the classification of susceptible (DZA315.16, F83005.5 and SA3780) and resistant (A17, DZA45,5, SA9048 and PRT180-A) lines (**Figure 5A**), with some variability in the colonization percentage among lines exhibiting low symptoms. Notably, the colonization of susceptible plants was always highest at 25°C and lowest at 28°C. Among the resistant lines, PRT180-A showed a noticeable colonization (percentage of 20%) at 28°C. For the hot-adapted mutant strain AS38 (**Figure 5B**), it appeared that lines DZA315.16, F83005.5, SA9048 and SA3780 were susceptible, with high colonization at 28°C. Significant differences in the plant responses to the wild type strain and its temperature-adapted mutant were observed: Line SA9048, which was partially resistant to V31-2 was highly susceptible to the mutant AS38 at 25°C and 28°C, and moderately susceptible at 20°C. Line DZA45.5 which was resistant to V31-2, could be considered as disease tolerant to AS38 at 25 and 28°C, since it was significantly colonized with highest colonization rate at 28°C despite low symptom scores (MSS<1.5). With the noticeable exception of A17, the resistant lines all exhibited higher colonization rates when infected with AS38, in particular at 28°C. Incidentally, these results also show that disease symptoms are always associated with plant colonization by the fungus, and thus not triggered by a toxin release by the fungus without plant colonization (Palmer et al., 2005).

### 3.3 Effect of *Verticillium* inoculation on fitness of *M. truncatula* is not proportional to temperature increase

As a first step to analyze the significance of the plants’ response to Verticillium at different temperatures, the fitness of plants inoculated with V31-2, and corresponding controls, at the three temperature regimes was studied. After four weeks, plants were transferred into the greenhouse where they continued to grow all under the same conditions. Pod number per plant, pod weight per plant and dry biomass of aerial parts per plant were assessed as proxy for fitness after six months. The germination rate of progeny seeds was 100% in all *M. truncatula* lines and for all conditions. Furthermore, the number of seeds per pod was not significantly different between lines and between infected and non-infected plants with an overall average number of five seeds per pods (data not shown). Hence the number of pods per plant seemed to be an accurate measure of total lifetime fitness.

**Figure 6:**
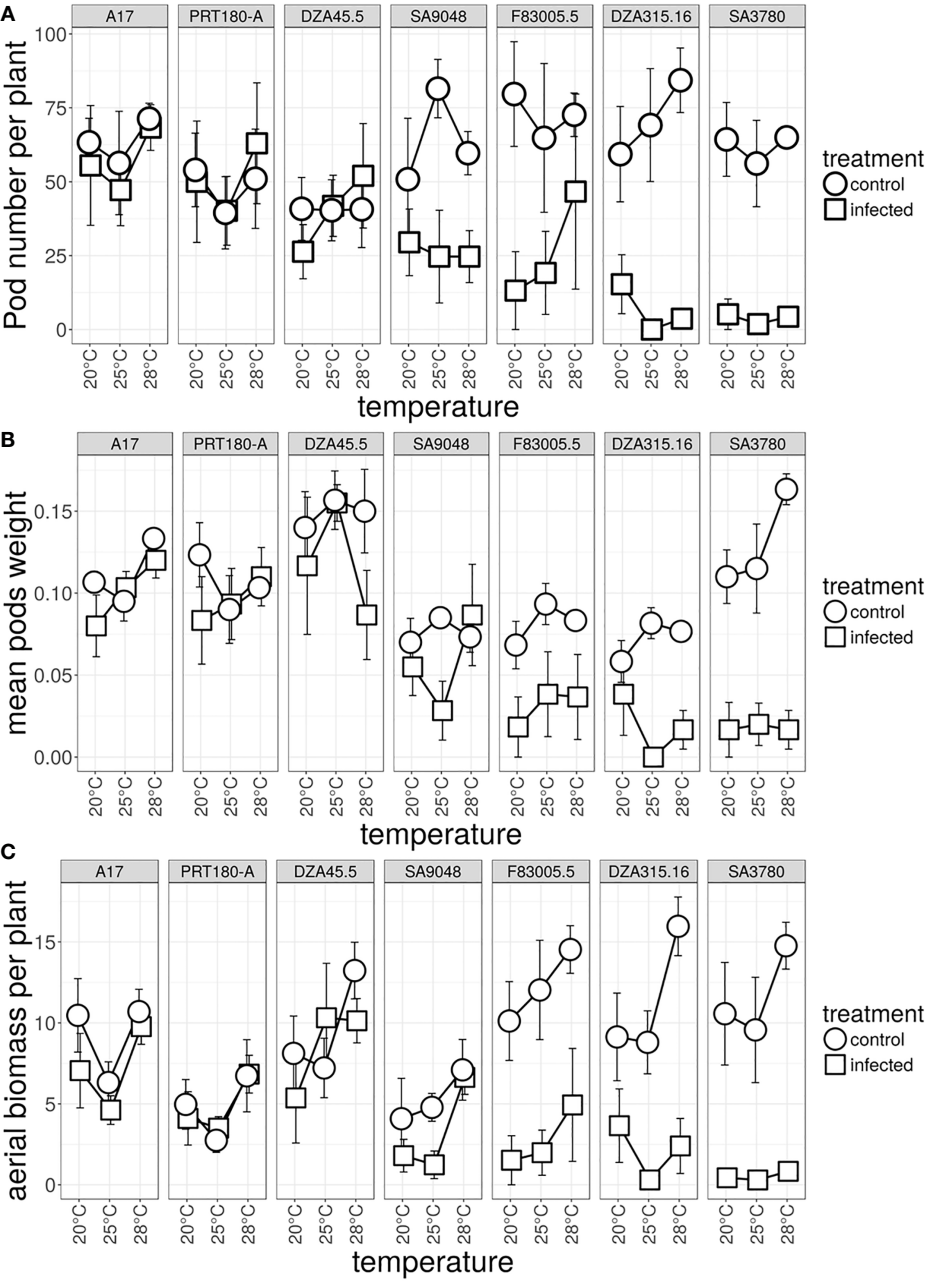
Effect of temperature on total lifetime fitness of *M. truncatula* inoculated, or not, with *Verticillium* strain V31-2. The *M. truncatula* lines A17, PRT180-A, DZA45.5, SA09048, F83005.5, DZA315,16 and SA03780 were mock-inoculated or root-inoculated with a spore suspension of *Verticillium* strain V31-2 and maintained at 20°C,25°C and 28°C during one month, then transferred to the greenhouse. The greenhouse conditions were identical for all plants. **(A)** pod number **(B)** pod weight and **(C)** weight of dry aerial mass were measured at the end of the plant’s cycle (approx. six months). Bars indicate standard error of the means of two independent experiments, with three blocks each.

Statistical analysis of the three fitness parameters showed that the effect of temperature observed during the first phase had disappeared (**Tables 3A–C**). However, a significant interaction line × treatment was observed, as expected for differences between susceptible and resistant lines in a compatible pathogenic interaction. Pod number was strongly affected in the susceptible lines F83005.5, DZA315.16 and SA3780, and moderately in the partially resistant line SA9048. This effect was not significant for the three remaining resistant lines A17, PRT180-A and DZA45.5 (**Figure 6A** and **Supplementary Table 9**). Pod weight and aerial biomass were significantly decreased by inoculation in the three susceptible lines. (**Figures 6B, C** and **Supplementary Tables 10**, **11**). As a general tendency, the fitness consequences of infection by V31-2 were always independent from the temperature overcame during the first month i.e. at early stages of infection.

Table 3Analysis of variance for fitness parameters.

**Table:** 

(A)					
Source of variation	Df	Sum Sq	Mean Sq	F value	Pr(>F)
Replicates	1	48	48	5.658	0.01853*
Block in Replicates	4	228.6	57.1	6.73	4.88e-05***
Line	6	192.2	32	3.773	0.00152**
Treatment	1	626	626	73.72	6.84e-15****
Temperature	2	6.5	3.3	0.384	0.68147
Line **×** Treatment	6	366.3	61.1	7.191	8.05e-07***
Line **×** Temperature	12	22.7	1.9	0.223	0.99715
Treatment **×** Temperature	2	1.7	0.8	0.097	0.90723
Line **×** Treatment **×** Temperature	12	70.1	5.8	0.688	0.76148
Residuals	163	1383.9	8.5

**Table:** 

(B)					
Source of variation	Df	Sum Sq	Mean Sq	F value	Pr(>F)
Replicates	1	0.00427	0.00427	2.247	0.135829
Block in Replicates	4	0.03201	0.008	4.209	0.002861**
Line	6	0.18931	0.03155	16.595	6.87E-15***
Treatment	1	0.0844	0.0844	44.391	3.94E-10***
Temperature	2	0.00212	0.00106	0.558	0.573266
Line **×** Treatment	6	0.04934	0.00822	4.325	0.000446***
Line **×** Temperature	12	0.0176	0.00147	0.772	0.678933
Treatment **×** Temperature	2	0.00025	0.00013	0.066	0.935926
Line **×** Treatment **×** Temperature	12	0.02368	0.00197	1.038	0.416893
Residuals	163	0.30991	0.0019

**Table:** 

(C)					
Source of variation	Df	Sum Sq	Mean Sq	F value	Pr(>F)
Replicates	1	1651.2	1651.2	108.091	< 2e-16***
Block in Replicates	4	99.7	24.9	1.632	0.169
Line	6	539.3	89.9	5.884	1.40E-05***
Treatment	1	1195.2	1195.2	78.243	1.44E-15***
Temperature	2	22.5	11.3	0.736	0.48
Line **×** Treatment	6	899.7	149.9	9.816	3.13E-09***
Line **×** Temperature	12	127.3	10.6	0.694	0.755
Treatment **×** Temperature	2	21.6	10.8	0.706	0.495
Line **×** Treatment **×** Temperature	12	155.2	12.9	0.847	0.602
Residuals	163	2490	15.3		

Seven *M. truncatula* accessions were infected by *V. alfalfae* strain V31-2 at 20°C, 25°C and 28°C for one month, then evaluated in a common garden experiment in green house. **(A)** ANOVA for pod number per plant (square-root transformation), **(B)** ANOVA for weight of pods per plant, **(C)** ANOVA for dry aerial biomass per plant.

*, **, and *** stands for 5%, 1% and 0.1% significance level respectively.

To test if the disease can spread *via* plants’ reproductive structures, Verticillium re-isolation experiments from pods and seeds from inoculated plants showed that the fungus was present inside the pods, but not inside the seeds, of susceptible lines, and absent from pods and seeds of resistant or partially resistant lines (data not shown), whatever the temperature.

### 3.4 Aggressiveness of VA31-2 and the temperature-adapted mutant on alfalfa

**Figure 7:**
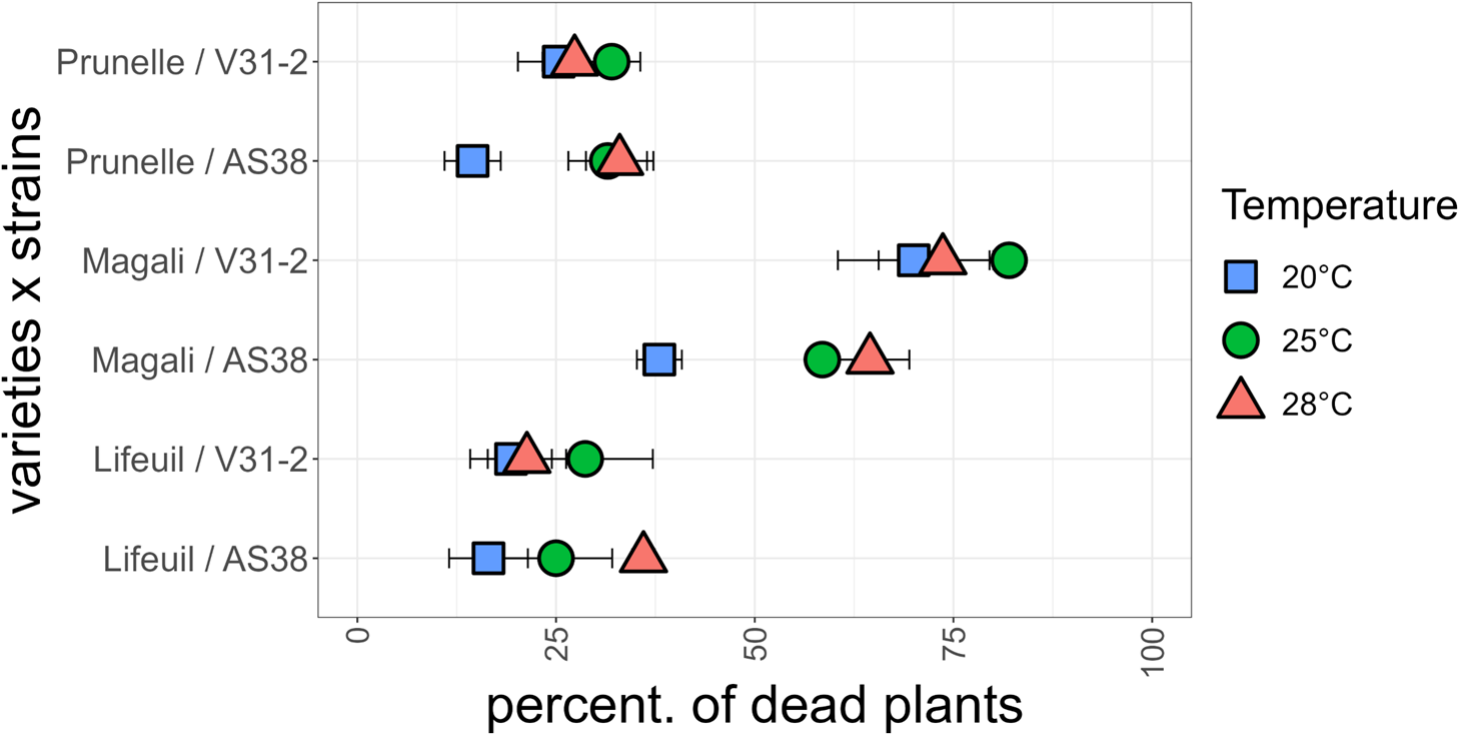
Mortality at the end of the experiment for three *M. sativa* varieties (Prunelle, Magali and Lifeuil) infected with *V. alfalfae* V31-2 and its derived mutant AS38, each at three temperatures. Data for each combination are from three independent experiments, each with 100 plants per variety. Bars indicate standard error of observed values.

The effect of temperature increase on Verticillium wilt and of potentially evolving new strains was studied in alfalfa (*M. sativa*), a cultivated crop phylogenetically close relative from the wild species *M. truncatula*. Three commercialized varieties were inoculated with the two *Verticillium* strains V31-2 and AS38, and their susceptibility was assessed by mortality rate at the end of the experiment (four weeks after inoculation). The combined analysis of deviance showed a very significant strain × temperature and strain × line interactions, but no line × temperature interaction, indicating that differences in disease response are not due to a differential response of the lines to temperature increase (**Table 4**). When the alfalfa cultivars were inoculated with strain V31-2 cultivar Magali was the most susceptible with 80% mortality, whereas Lifeuil and Prunelle were partially resistant with not more than 30% mortality (**Figure 7**). The highest mortality was observed at 25°C, and the lowest at 28°C (**Table 4**). When the alfalfa cultivars were inoculated with strain AS38, cultivar Magali was the most susceptible with 60% and 65% mortality at 25°C and 28°C respectively, whereas Lifeuil and Prunelle were partially resistant with not more than 40% mortality (**Figure 7**). The highest mortality was observed at 25°C and 28°C, and the lowest at 20°C. These results confirm that temperature is a significant determinant of *Verticillium* aggressiveness towards *M. sativa*. They also demonstrate that AS38, a pathogenic strain which was initially adapted on *M. truncatula* for higher temperatures, is able to infect another species *M. sativa* with the same optimum temperature for disease onset (28°C).

**Table 4:** Analysis of deviance for the percentage of dead plants at 28 dpi, for three *M. sativa* cultivars. 100 plants per variety were infected by *V. alfalfae* strains V31-2 and AS38, at 20°C, 25°C and 28°C, in three independent replicates and two blocks per replicate.

	Df	Deviance	Resid. Df	Resid. Dev	Pr(>Chi)
null model			44	920.21	
Cultivar	2	700.81	42	219.4	< 2.2 10^-16^ ***
Temperature	2	61.17	40	158.23	5.215 10^-14^ ***
Strain	1	26.06	39	132.17	3.311 10^-07^ ***
Cultivar **×** Temperature	4	0.99	35	131.18	0.9111
Cultivar **×** Strain	2	52.28	33	78.9	4.441 10^-12^ ***
Temperature **×** Strain	2	31.41	31	47.48	1.510 10^-07^ ***

Df, degree of freedom; Deviance, reduction in residual deviance due to the added factor; Resid. Df, degree of freedom for residual terms; Resid. Dev, residual deviance; Pr(>Chi), probability of getting observed Chi Squared-values under the null hypothesis. Terms are added sequentially (first to last).

*, **, and *** stands for 5%, 1% and 0.1% significance level respectively.

## 4 Discussion

Temperature has long been recognized as a major determinant of the plant ‘disease triangle’, together with a virulent pathogen and a permissive host (Agrios, 2005). We focused on legumes because of their importance for ecosystems and as major sustainable suppliers of food and feed. We also focused on the detailed analysis of one temperature-adapted mutant of a root pathogen as a possible paradigm of future events.

Below 28°C, temperature increase was shown to favor mycelium growth and sporulation of twelve *Verticillium* strains from various host plants and geographic origins. The two parameters were not always correlated. For example, temperature increase (from 20°C to 28°C) improved mycelium growth of Vdp5 but reduced its sporulation. On average, the optimum for sporulation was lower than for mycelium growth, a situation already described for several fungi (e.g. Cooperman, 1986; Michailides, 1987). Interestingly, the AS38 mutant strain grew significantly less and had low sporulation capacity at 28°C compared to 25°C, while it was the most virulent and aggressive strain *in planta* at this temperature. This confirms that *in vitro* fitness’ evaluation may not be an accurate predictor of pathogenicity (Korolev et al., 2000). However, it is possible that temperatures inside the plant may be lower than in the environment. Thermoregulation in plants is described in several cases, usually for maintaining temperature in leaves or flowers (Willey, 2015) but the interaction of plant thermoregulation with disease responses, and its influence in putatively mitigating some effects of climate change is still unknown.

We experimentally adapted the *V. alfalfae* strain V31-2 to higher aggressiveness and virulence at 28°C, using successive rounds of mutagenesis and re-isolation from infected tissues in a susceptible *M. truncatula* line. Experimental evolution experiments are routinely described for plant pathogenic bacteria (Lenski et al., 1991; Marchetti et al., 2010; Guidot et al., 2014; Manriquez et al., 2021) but more rarely for fungi (Dettman et al., 2008; Kohn et al., 2008; Jeon et al., 2013; Fisher and Lang, 2016). The adaptation of the original V31-2 strain was efficiently achieved in three rounds, and provided us with the AS38 strain which was more aggressive at 28°C on all *M. truncatula* lines except on line A17. The increase in aggressiveness of AS38 at higher temperatures seems to be associated with fitness cost *in vitro* with less growth and sporulation than its wild type (Delmas et al., 2016; Esterio et al., 2021). On alfalfa, strain AS38 also infected best at 28°C, but displayed lower aggressiveness at all temperatures when compared to its wild-type V31-2. This might be due to some specificity of adaptation on *M. truncatula*. This experiment clearly exemplifies that climate change may lead to the rapid appearance of adapted fungal pathogens, with conserved capacities to infect different hosts species. It also highlights the importance of wild species as reservoirs for crop diseases (Parker and Gilbert, 2004; Awasthi et al., 2015; Yazdkhasti et al., 2021). Christen and French (1982) found that a range of *V. alfalfae* strains (*V. albo-atrum* isolated from alfalfa) from western Canada and north-western USA had an optimal temperature in culture of 25°C. Comparisons of aggressiveness in the UK and the USA using several European and North American alfalfa strains and temperature ranges of 17–30°C and 12–21°C, respectively, showed that disease severity was greater at the highest temperatures tested in each country (Christen et al., 1983). Altogether, the experimental results are in agreement with the observed southwards move, in less than 25 years, of *V. alfalfae* from Canada to Southern California (Aubé and Sackston, 1964; Erwin et al., 1988). The putative rapid adaptation of a major crop pathogen in a related wild species may be relevant for providing concepts to build new policies for agriculture, with consequences on crop protection strategies in a context of global warming. The genomic and phenotypic characterization of AS38 and other highly aggressive strains at 28°C strains will be the next step to understand adaptative changes related to temperature and host range.

Early disease symptoms broadly reflected fitness of the lines at later stages, with susceptible, partially resistant and resistant lines, confirming that strong Genome×Genome interactions are translated at total lifetime fitness level. As a counter-example, the line SA9048 was resistant to strain V31-2 whatever the temperature, as suggested by low early symptom scores and low plant colonization rate (**Figure 5A**), but its pod number per plant was significantly reduced compared to mock-inoculated control at later stages (**Figure 6A** and **Supplementary Table 8**). The discrepancy between early resistance and late susceptibility in this particular case may be due to a slow progress of the disease in that line, or to fitness cost of plant’s defense reactions. In summary, moderate temperature increase (+3°C to +5°C with respect to optimum) has an effect on early disease symptoms and plant colonization. If the plants are susceptible or tolerant, this effect is later masked by the consequence of the disease development, with all fitness proxies strongly affected. If the plants are resistant, the moderate range of temperature surveyed (20°C-28°C) has little or no effect on fitness proxies. For soil-borne pathogens our results thus suggest that temperature increase due to climate change will increase host susceptibility at early stages.

Statistical analysis of disease severity symptoms (MSS and AUDPC) evidences a clear effect of temperature on aggressiveness, combined with a strain and line effect. Even if the number of *M. truncatula* lines and *Verticillium* strains surveyed in this analysis is modest, this trend towards an increased virulence of hot-adapted strains is challenging in terms of forecasting disease epidemics and deserves to be studied with a larger panel. The hot-adapted AS38 strain developed a new virulence towards the line SA9048 at all temperatures, with maximum aggressiveness at 28°C. Thus, the temperature increase due to climate change may also lead to emergence of new diseases, by selecting for new compatible interactions as a consequence of pathogen micro-evolution. This phenomenon might also be related to temperature-dependent modifications of the plant’s response. The effect of temperature increase on monogenic qualitative resistance is illustrated by the interaction of tobacco and tobacco mosaic virus, a typical gene-for-gene interaction where the *N* resistance gene product interacts with the virus helicase. *N-*dependent resistance is abolished at temperatures above 28°C (Samuel, 1931). The transcription of the *N* gene is temperature-dependent and does not occur at restrictive temperature (Takabatake et al., 2006). Another temperature effect but opposite is illustrated by the temperature-dependent partial resistance against *Puccinia striiformi*, the causal agent of *s*tripe rust in wheat, in which the gene *Yr36* (WKS1) confers non race-specific resistance at relatively high temperatures (25–35°C) in agreement with higher expression levels at these temperatures (Fu et al., 2009). The fact that resistance to *Verticillium* in *M. truncatula* is a QDR dependent on several loci might explain that temperature effects are more subtle.

The increase of plant colonization by the AS38 strain at 28°C is noticeable (**Figure 5B**), with exception of line A17 which was fully resistant to both AS38 and V31-2 at all temperatures. We also identify situations where plants with few or no symptoms exhibit high levels of colonization such as DZA45.5 infected with AS38 (**Figure 5B**). In tomato, resistance against *Verticillium* largely depends on the isolation of the fungus in contained parts of the xylem followed by subsequent elimination of the fungus (Fradin and Thomma, 2006). This is also the case for the full resistance of A17 which takes place in roots (Toueni et al., 2016). The influence of temperature on mechanisms of containment or elimination by the plant and on stresses pathogen fitness inside the plant’s tissues needs to be analyzed more deeply in order to understand the influence of temperature on quantitative disease resistances. The mechanisms that account for tolerance are still debated (Robb, 2007).

In conclusion, this study clearly establishes that temperature increase may favor disease development caused by the soil-borne pathogen *V. alfalfae* and that the fungus is able to quickly adapt to temperature increase, and may even become more virulent and aggressive. This is in agreement with reports that Verticillium wilt of rape seed caused by *V. longisporum* has occurred more frequently in recent warmer years (Siebold and von Tiedemann, 2012) and that colonization of plants by the fungus may be advanced in time, potentially leading to higher inoculum densities after harvest under future warming (Siebold and von Tiedemann, 2013). Based on these reports and our findings, warmer temperature may thus increase the overall incidence of Verticillium wilt in agriculture and natural ecosystems. We propose that standard inoculation tests for assessing Verticillium resistance in alfalfa which are usually performed at 20°C (Molinéro-Demilly et al., 2007) should also include assays at the higher temperature of 25°C which is more appropriate to detect susceptibility of the host plant. It will also be necessary to assess the consequences of putative pathogen adaptation and modifications of host responses, by translating our results from glasshouse to the fields and ecosystems. This might include studies of the combined effect of increasing temperature, rising CO_2_ and altered moisture. Taking into account the particularities of legume biology, further work is also needed to understand the putative interactions with root symbioses.

## Data availability statement

The raw data supporting the conclusions of this article will be made available by the authors, without undue reservation.

## Author contributions

AS performed all experiments with collaboration of MM. LG and CB contributed to the experimental designs and performed some statistical analysis. MR contributed to data analysis. CB, LG and MR wrote the manuscript. All authors contributed to the article and approved the submitted version.
