# Supplementary material for: Temperature increase modifies susceptibility to Verticillium wilt in Medicago spp and may contribute to the emergence of more aggressive pathogenic strains
Source: Front Plant Sci. 2023 Feb 14;14:1109154. doi: 10.3389/fpls.2023.1109154 (PMC9972977; doi:10.3389/fpls.2023.1109154)
Supplement: Supplementary file 1 [file DataSheet_1.pdf]

Supplementary Material

Temperature increase modifies susceptibility to Verticillium wilt in *Medicago spp* and may contribute to the emergence of more aggressive pathogenic strains

Abed Al Latif Sbeiti[1], Mélanie Mazurier[1], Cécile Ben[1,2], Martina Rickauer[1], Laurent Gentzbittel[1,2*]

*** Correspondence:**
Laurent Gentzbittel
l.gentzbittel@skoltech.ru

1 Supplementary Data

2 Supplementary Figures and Tables

2.1 Supplementary Figures

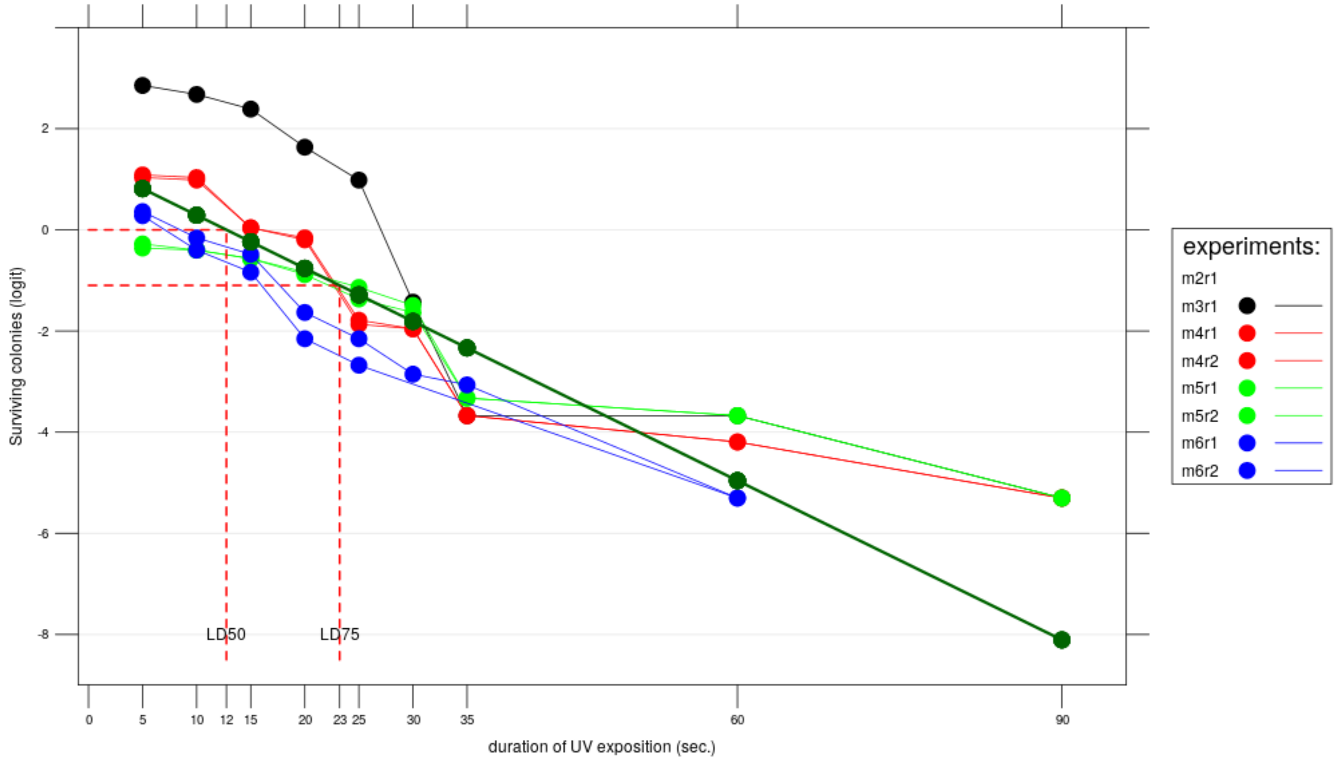

Supplementary Figure S1. Determination of LD50 for UV mutagenesis of *Verticillium alfalfae* strain V31-2. Hundred spores were spotted on PDA plates and subjected to UV irradiation for several durations (in seconds) as described in Buxton and Hastie (1962). After one week of growth, the number of surviving spores was assessed. Seven independent replicates of the experiment were used to fit a generalized linear model using a logit link, for the proportion of surviving spores as a function of time of UV exposition. LD50 (12 sec.) and LD75 were determined using the model fit.

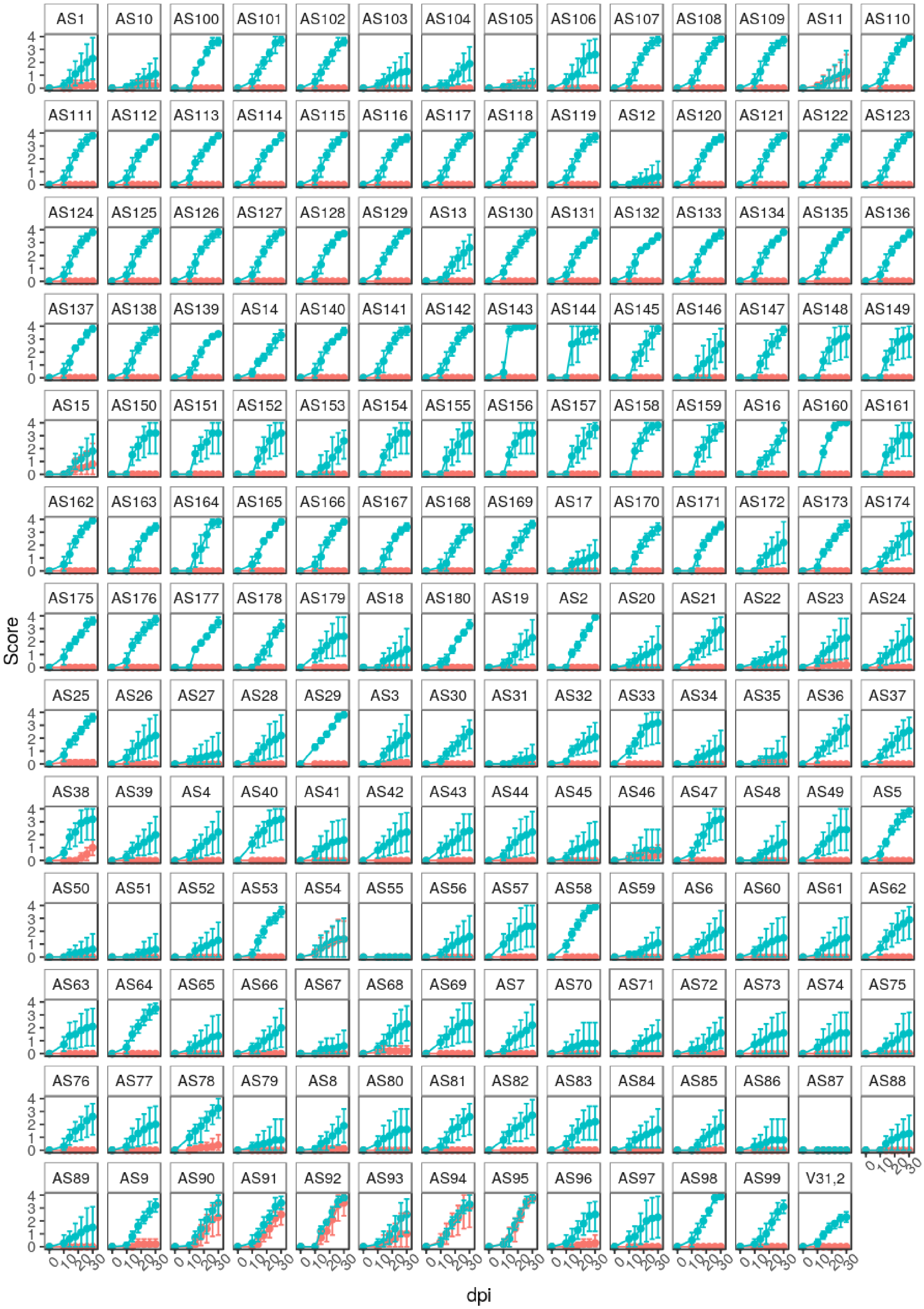
AS1
AS10
AS100
AS101
AS102
AS103
AS104
AS105
AS106
AS107
AS108
AS109
AS11
AS110
AS111
AS112
AS113
AS114
AS115
AS116
AS117
AS118
AS119
AS12
AS120
AS121
AS122
AS123
AS124
AS125
AS126
AS127
AS128
AS129
AS13
AS130
AS131
AS132
AS133
AS134
AS135
AS136
AS137
AS138
AS139
AS14
AS140
AS141
AS142
AS143
AS144
AS145
AS146
AS147
AS148
AS149
AS15
AS150
AS151
AS152
AS153
AS154
AS155
AS156
AS157
AS158
AS159
AS16
AS160
AS161
AS162
AS163
AS164
AS165
AS166
AS167
AS168
AS169
AS17
AS170
AS171
AS172
AS173
AS174
AS175
AS176
AS177
AS178
AS179
AS18
AS180
AS19
AS2
AS20
AS21
AS22
AS23
AS24
AS25
AS26
AS27
AS28
AS29
AS3
AS30
AS31
AS32
AS33
AS34
AS35
AS36
AS37
AS38
AS39
AS4
AS40
AS41
AS42
AS43
AS44
AS45
AS46
AS47
AS48
AS49
AS5
AS50
AS51
AS52
AS53
AS54
AS55
AS56
AS57
AS58
AS59
AS6
AS60
AS61
AS62
AS63
AS64
AS65
AS66
AS67
AS68
AS69
AS7
AS70
AS71
AS72
AS73
AS74
AS75
AS76
AS77
AS78
AS79
AS8
AS80
AS81
AS82
AS83
AS84
AS85
AS86
AS87
AS88
AS89
AS9
AS90
AS91
AS92
AS93
AS94
AS95
AS96
AS97
AS98
AS99
V31,2
Score
0 1 2 3 4
0 10 20 30
dpi

Supplementary Figure S2. Disease symptoms caused by 180 V31.2 mutants on two contrasted *M. truncatula* lines. Plants of lines A17 (resistant, in orange) and F83005.5 (susceptible, in blue) were root-inoculated with a spore suspension of V31.2 and monospore isolates of its derived mutants, and maintained at 28°C for 4 weeks. Symptoms were scored regularly on a scale from 0 to 4. The values are means from five plants and bars indicate standard error of observed values for symptoms caused by 180 randomly chosen mutants and V31-2 wild type. As expected, a large majority of mutants exhibits increased aggressiveness on F83005.5 line at 28°C. The AS38 mutant presents also increased aggressiveness on A17 line.

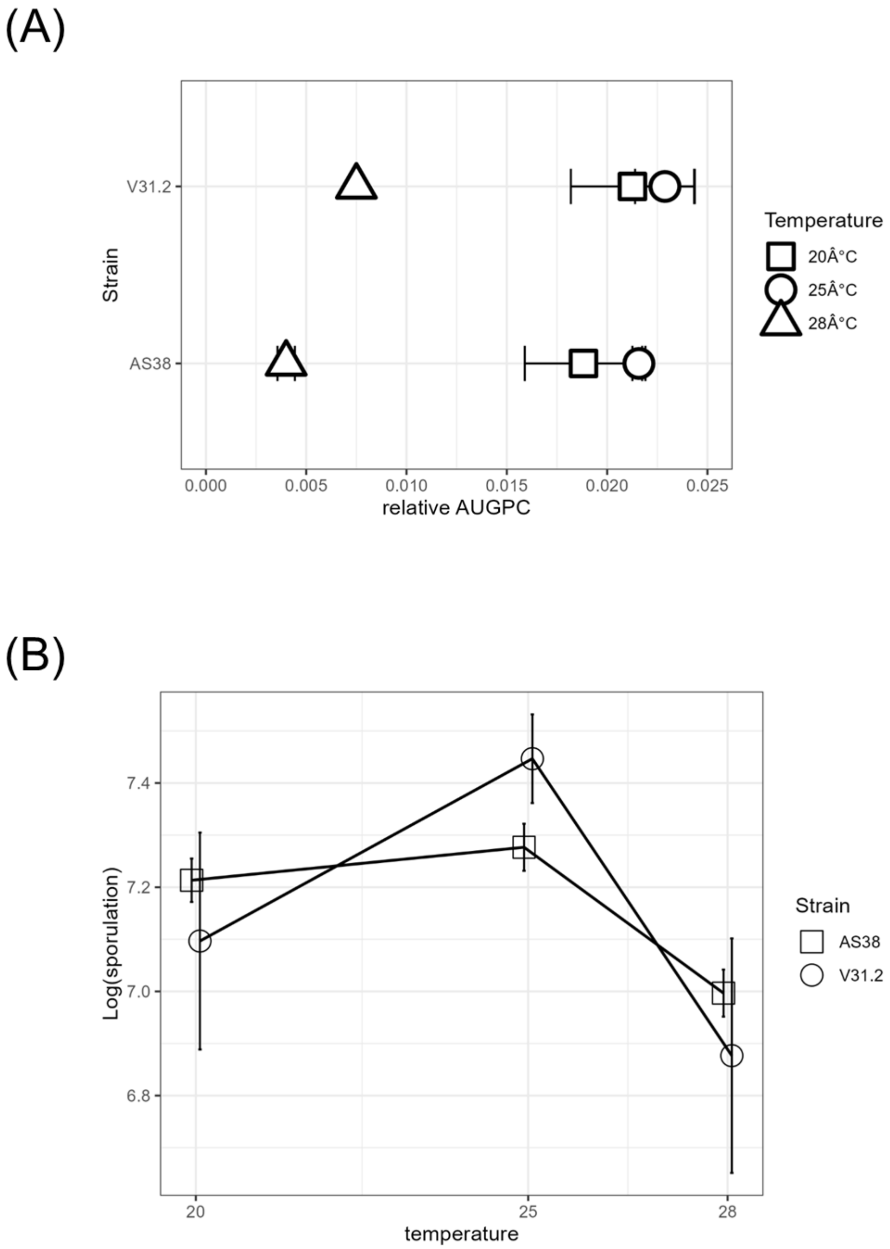

Supplementary Figure S3. Effect of temperature on *in vitro* growth **(A)** and sporulation **(B)** of *Verticillium* alfalfa strain V31.2 and its temperature-adapted mutant AS38. (**A**) strains were grown on PDA medium in Petri dishes at 20°C, 25°C and 28°C. Radial growth was measured during 14 days and expressed as Area Under Growth Progress Curve (AUGPC). (**B**) Conidia released into distilled water were counted with a hematocytometer. Each point shows the mean values of three independent experiments, with two Petri dishes each. Bars indicate standard error of observed values.

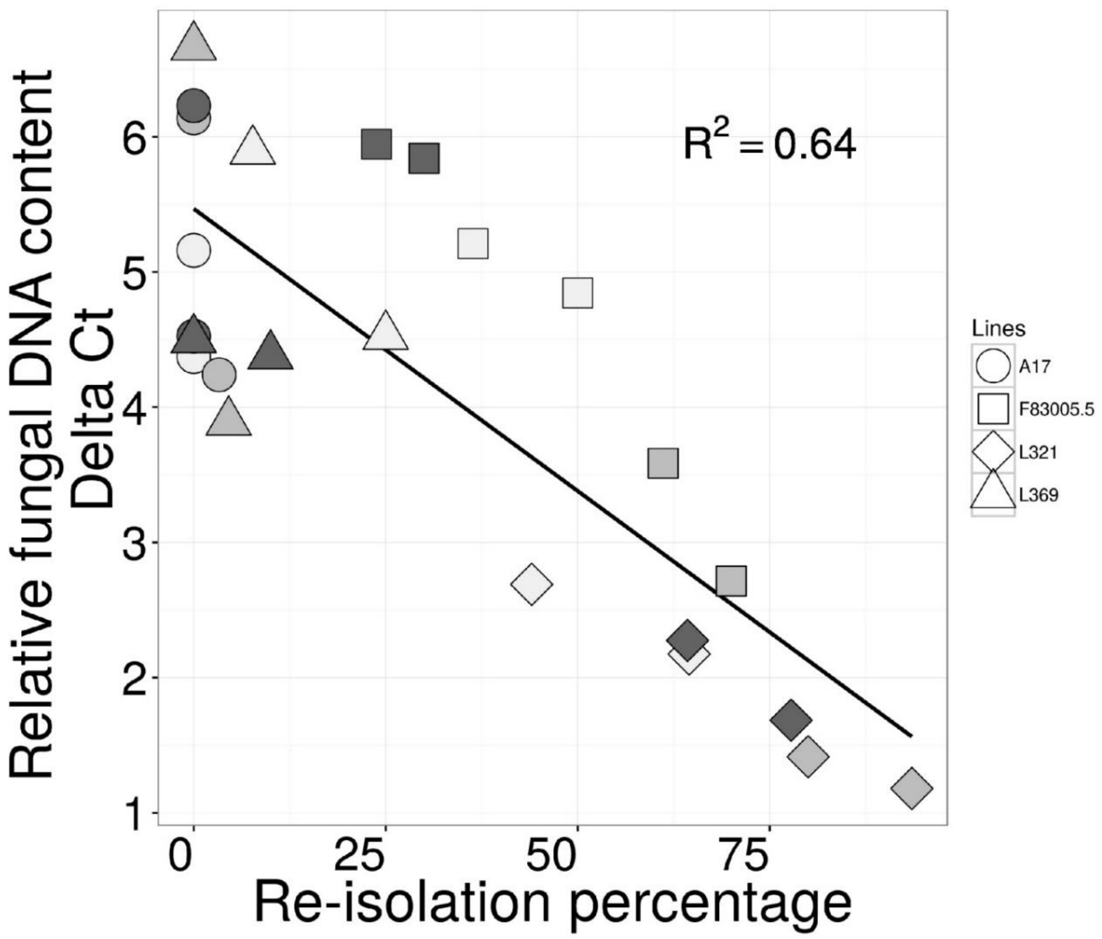

Supplementary Figure S4. Quantification of *Verticillium in planta* by qPCR compared to re-isolation assay. Fungal DNA is expressed as ΔCt (Ct V31-2 DNA – Ct *M. truncatula* DNA). Two independent experiments were performed at 20°C (open symbols), 25°C (gray symbols) and 28°C (blacksymbols) respectively, and are shown individually. DNA was extracted at 10 dpi from the first leaf. *Verticillium* re-isolation from stem sections was performed at the end of the experiment, at 30 dpi. Values show the means of ten plants.

2.2 Supplementary Tables

Line	Verticillium wilt resistance rating [a]	Country	HapMap code [b]
A17	resistant	Spain[c]	Reference genome
PRT180-A	partially resistant	Portugal	HM038
DZA45.5	partially resistant	Algeria	HM004
SA9048	partially resistant	Libya	HM041
F83005.5	susceptible	France	HM006
DZA315.16	susceptible	Algeria	HM005
SA3780	very susceptible	Italy	HM040

[a] Based on published literature from V31-2 inoculation experiments carried out at 20°C (Ben *et al.* 2013; Negahi *et al.* 2013) and this study. [b] http://www.medicagohapmap.org. [c] Based on Gentzbittel *et al*. 2019.

(B)

Variety	Seed company	Verticillium wilt resistance rating [a]	Protein content
Magali	INRA	susceptible	Moderately good
Lifeuil	R2N	resistant	Good
Prunelle	Florimond Desprez	resistant	Good

[a] Based on published literature from V31-2 inoculation experiments carried out at 20°C (Molinéro-Demilly et al. 2007).

Supplementary Table 1: Plant material used in this study. **(A)** *M. truncatula* genotypes, their resistance phenotype towards *V. alfafae* V31-2 strain and their geographical origin. **(B)** Alfalfa varieties, company which provided seeds, Verticillium field resistance and protein content as indicated by seed companies.

Strain	Species*	Isolated from	Origin
V31-2[a]	*alfalfae*	Alfalfa	France
VA1[b]	*non-alfalfae*	Potato	Canada
JR2[b]	*dahliae*	Tomato	Unknown
LPP0323[c]	*non-alfalfae*	Potato	USA
VA2[d]	*alfalfae*	Alfalfa	Germany
Vd89[d]	*dahliae*	Pea	Germany
Vdp1[e]	*dahliae*	Potato	Israel
Vdp2[e]	*dahliae*	Potato	Israel
Vdp3[e]	*dahliae*	Potato	Israel
Vdp5[e]	*dahliae*	Potato	Israel
V198[f]	*dahliae*	Cotton	Spain
V200[f]	*dahliae*	Cotton	Spain

* based on molecular classification (this work) as described by Inderbitzin *et al.* (2011) or as reported by the authors. The strains were kindly provided by: [a] Barenbrug , [b] B. Thomma, [c] A. von Tiedemann, [d] G. Bause, [e] L. Zror, [f] R. Jimenez-Diaz

Supplementary Table 2 : *Verticillium spp.* strains used in this work and their geographical origin.

	Df	SS	MS	F value	Pr(>F)
replicate	2	3.82E-05	1.91E-05		
strain	11	0.0004847	4.41E-05	13.378	< 2e-16 ***
temperature	2	0.0004912	2.46E-04	74.561	< 2e-16 ***
strain x temperature	22	0.0008683	3.95E-05	11.982	< 2e-16 ***
residuals	178	0.0005863	3.29E-06		

Supplementary Table 3 : Analysis of variance of Area Under the Growth Progression Curve for 12 *Verticillium* strains depending on temperature. Df : degree of freedom ; SS : sum of squares ; MS : mean square ; Pr(>F) : probability of getting observed F-values under the null hypothesis.

	Df	Deviance	Resid. Df	Resid. Dev	Pr(>Chi)
null model			107	2616002496	
replicate	2	105174629	105	2510827867	< 2.2e-16 ***
strain	11	1790291337	94	720536530	< 2.2e-16 ***
temperature	2	436687319	92	283849211	< 2.2e-16 ***
strain x temperature	22	102148956	70	181700255	< 2.2e-16 ***

Supplementary Table 4 : Analysis of deviance of sporulation capacity for 12 *Verticillium* strains depending on temperature. Df : degree of freedom ; Deviance : reduction in deviance due to the added factor; Pr(>Chi) : probability of getting observed ChiSquared-values under the null hypothesis. Terms are added sequentially (first to last) in the Poisson regression.

Factor	Chi-Square	d.f.	P
replicate	14.91	2	0.0006
Strain	423.36	132	<.0001
All Interactions with Strain	355.51	121	<.0001
Temperature	403.28	96	<.0001
All Interactions with Temperature	262.82	94	<.0001
Line	487.34	108	<.0001
All Interactions with Line	334.31	105	<.0001
Strain x Temperature	223.74	88	<.0001
Strain x Line	302.74	99	<.0001
Temperature x Line	184.75	72	<.0001
Strain x Temperature x Line	137.47	66	<.0001
TOTAL	589.61	97	<.0001

Supplementary Table 5 : Analysis of deviance of proportional-odds model for Maximum Symptom Scores, depending on 12 naturally-occuring *Verticillium* strains, four *M. truncatula* lines and three levels of temperature. Chi-Square : reduction in deviance due to the added factor; d.f. : degree of freedom; P : probability of getting observed ChiSquared-values under the null hypothesis. Terms are added sequentially (first to last)

Strain	Temperature	Line	group
JR2	28°C	DZA45.5	a
	28°C	A17	ab
	25°C	F83005.5	ab
	28°C	DZA315.16	abc
	28°C	F83005.5	abc
	20°C	F83005.5	abc
	25°C	A17	bcd
	20°C	DZA45.5	cd
	20°C	A17	cd
	25°C	DZA315.16	cd
	20°C	DZA315.16	d
	25°C	DZA45.5	d
LPP0323	Temperature	Line	group
	25°C	F83005.5	a
	28°C	DZA45.5	a

	25°C	DZA45.5	a
	28°C	F83005.5	a
	28°C	A17	ab
	28°C	DZA315.16	ab
	25°C	A17	abc
	20°C	F83005.5	abc
	20°C	DZA45.5	bcd
	25°C	DZA315.16	cde
	20°C	A17	de
	20°C	DZA315.16	e
V192	Temperature	Line	group
	28°C	F83005.5	a
	28°C	DZA45.5	a
	25°C	DZA45.5	a
	28°C	DZA315.16	a
	28°C	A17	ab
	20°C	F83005.5	ab

	25°C	A17	bc
	25°C	F83005.5	bc
	20°C	DZA45.5	cd
	25°C	DZA315.16	cd
	20°C	A17	cd
	20°C	DZA315.16	d
V200	Temperature	Line	group
	28°C	DZA45.5	a
	28°C	F83005.5	a
	28°C	DZA315.16	ab
	25°C	F83005.5	abc
	25°C	DZA45.5	abc
	28°C	A17	abcd
	20°C	F83005.5	bcde
	20°C	DZA45.5	cde
	25°C	A17	def
	20°C	DZA315.16	efg

	20°C	A17	fg
	25°C	DZA315.16	g
V31-2	Temperature	Line	group
	20°C	A17	a
	25°C	A17	ab
	28°C	DZA45.5	ab
	28°C	A17	abc
	25°C	DZA45.5	abc
	20°C	DZA45.5	bcd
	28°C	F83005.5	cde
	28°C	DZA315.16	cde
	20°C	DZA315.16	de
	20°C	F83005.5	e
	25°C	F83005.5	e
	25°C	DZA315.16	e
VA1	Temperature	Line	group
	25°C	DZA45.5	a

	28°C	DZA45.5	a
	25°C	F83005.5	ab
	28°C	F83005.5	ab
	25°C	A17	abc
	20°C	F83005.5	abc
	20°C	DZA45.5	bc
	28°C	A17	cd
	25°C	DZA315.16	de
	28°C	DZA315.16	de
	20°C	A17	e
	20°C	DZA315.16	e
VA2	Temperature	Line	group
	25°C	DZA45.5	a
	28°C	F83005.5	a
	25°C	A17	a
	28°C	DZA45.5	a
	25°C	F83005.5	a

	20°C	F83005.5	a
	20°C	A17	a
	20°C	DZA45.5	a
	28°C	DZA315.16	a
	28°C	A17	a
	25°C	DZA315.16	b
	20°C	DZA315.16	b
Vd89	Temperature	Line	group
	28°C	F83005.5	abc
	28°C	DZA45.5	abc
	20°C	F83005.5	abc
	25°C	A17	abc
	25°C	DZA45.5	a
	25°C	F83005.5	a
	28°C	A17	a
	20°C	A17	ab
	28°C	DZA315.16	abc

	20°C	DZA45.5	abc
	25°C	DZA315.16	bc
	20°C	DZA315.16	c
Vdp1	Temperature	Line	group
	25°C	F83005.5	a
	28°C	DZA45.5	a
	28°C	F83005.5	ab
	28°C	A17	ab
	25°C	DZA45.5	abc
	28°C	DZA315.16	abcd
	20°C	A17	bcd
	20°C	DZA45.5	bcd
	25°C	A17	bcd
	25°C	DZA315.16	cd
	20°C	DZA315.16	d
	20°C	F83005.5	d
Vdp2	Temperature	Line	group

	25°C	F83005.5	a
	28°C	A17	a
	28°C	F83005.5	a
	28°C	DZA315.16	ab
	28°C	DZA45.5	ab
	20°C	F83005.5	ab
	25°C	DZA45.5	abc
	20°C	DZA45.5	bcd
	25°C	A17	bcd
	20°C	A17	cde
	20°C	DZA315.16	de
	25°C	DZA315.16	e
Vdp3	Temperature	Line	group
	25°C	DZA45.5	abc
	28°C	F83005.5	abc
	28°C	A17	a
	28°C	DZA315.16	a

	28°C	DZA45.5	a
	25°C	F83005.5	a
	25°C	A17	ab
	20°C	F83005.5	ab
	20°C	DZA45.5	bc
	25°C	DZA315.16	bc
	20°C	A17	c
	20°C	DZA315.16	c

Vdp5	Temperature	Line	group
	25°C	F83005.5	ab
	25°C	A17	ab
	28°C	DZA45.5	a
	28°C	F83005.5	a
	20°C	A17	a
	20°C	DZA45.5	a
	25°C	DZA45.5	a

28°C	A17	a
20°C	F83005.5	a
28°C	DZA315.16	ab
25°C	DZA315.16	b
20°C	DZA315.16	ab

Supplementary Table 6. Multiple comparisons of aggressiveness of 12 Verticillium strains on four lines of *Medicago truncatula* at different temperatures. For each strain, data were analysed using proportional-odds models with Temperature and Line effect.

Temperature	Strain	Line	group
20°C	V31-2	A17	a
	AS38	A17	a
	V31-2	DZA45.5	ab
	AS38	PRT180-A	ab
	V31-2	PRT180-A	ab
	AS38	DZA45.5	abc
	V31-2	SA09048	bcd
	V31-2	F83005.5	cde
	AS38	SA09048	de
	AS38	F83005.5	de
	V31-2	DZA315.16	de
	AS38	DZA315.16	e
	V31-2	SA03780	e
	AS38	SA3780	e
	Strain	Line	group

25°C	AS38	A17	a
	V31-2	A17	a
	AS38	PRT180-A	a
	V31-2	PRT180-A	ab
	AS38	DZA45.5	ab
	V31-2	SA9048	b
	V31-2	DZA45.5	b
	AS38	DZA315.16	c
	V31-2	F83005.5	c
	AS38	SA9048	c
	V31-2	DZA315.16	c
	AS38	F83005.5	c
	V31-2	SA3780	c
	AS38	SA3780	c
	Strain	Line	group
28°C	V31-2	A17	a
	AS38	A17	a

	V31-2	SA9048	a
	V31-2	PRT180-A	ab
	AS38	PRT180-A	ab
	V31-2	DZA45.5	ab
	AS38	DZA45.5	ab
	V31-2	F83005.5	b
	V31-2	DZA315.16	bc
	V31-2	SA3780	c
	AS38	DZA315.16	c
	AS38	SA9048	c
	AS38	F83005.5	c
	AS38	SA3780	c

Supplementary Table 7. Multiple comparisons of aggressiveness of *Verticillium* strain V31-2 and mutant strain AS38 on seven lines of *Medicago truncatula* at different temperatures. For each strain, data were analysed using proportional-odds models with Strain and Line effect.

	Df	Deviance	Resid. Df	Resid. Dev	Pr(>Chi)
null model			71	5675.9	
strain	1	278.6	70	5397.3	< 2.2e-16 ***
accession	3	4942.2	67	455.1	< 2.2e-16 ***
temperature	2	22.7	65	432.4	1.174e-05 ***
strain x accession	3	203.6	62	228.8	< 2.2e-16 ***
strain x temperature	2	107.8	60	121.0	< 2.2e-16 ***
accession x temperature	6	61.9	54	59.1	1.855e-11 ***
strain x accession x temperature	6	43.4	48	15.8	9.885e-08 ***

Supplementary Table 8 : Analysis of deviance of re-isolation rate of V31.2 or AS38 *V. alfalfae* strains assessed on four *M. truncatula* lines at three temperatures 20C, 25C and 28C. Df : degree of freedom ; Deviance : reduction in deviance due to the added factor; Pr(>Chi) : probability of getting observed ChiSquared-values under the null hypothesis. Terms are added sequentially (first to last).

	20°C		25°C		28°C	
Line	C	I	C	I	C	I
A17	63 *a*	56 *a*	56 *a*	47 *a*	71 *a*	58 *a*
PRT180-A	54 *ab*	50 *ab*	40 *ab*	40 *ab*	51 *ab*	63 *ab*
DZA45.5	41 *ab*	26 *ab*	40 *ab*	42 *ab*	41 *ab*	52 *ab*
SA9048	51 *a*	30 *bc*	82 *a*	25 *bc*	60 *a*	25 *bc*
F83005.5	80 *a*	13 *cd*	65 *a*	19 *cd*	73 *a*	47 *cd*
DZA315.16	59 *a*	15 *d*	69 *a*	0 *d*	84 *a*	4 *d*
SA3780	64 *a*	5 *d*	56 *a*	2 *d*	65 *a*	4 *d*

Supplementary Table 9. Net effect of combined Verticillium infection with V31-2 strain and temperature at infection, on number of pods per plant for seven *M. truncatula* lines. 20°C, 25°C and 28°C are the temperatures of infection and first four weeks of growth. C : control condition, I : inoculated. Different letters indicate a significant difference at P<0,05.

	20°C		25°C		28°C	
Line	C	I	C	I	C	I
A17	0.11 *bcd*	0.08 *bcd*	0.1 *bcd*	0.10 *bcd*	0.10 *bcd*	0.10 *bcd*
PRT180-A	0.12 *bcd*	0.08 *bcde*	0.1 *bcd*	0.09 *bcde*	0.10 *bcd*	0.11 *bcde*
DZA45.5	0.14 *a*	0.12 *ab*	0.16 *a*	0.16 *ab*	0.15 *a*	0.09 *ab*
SA9048	0.07 *cde*	0.06 *ef*	0.09 *cde*	0.03 *ef*	0.07 *cde*	0.09 *ef*
F83005.5	0.07 *bcde*	0.02 *f*	0.1 *bcde*	0.04 *f*	0.08 *bcde*	0.04 *f*
DZA315.16	0.05 *de*	0.04 *f*	0.08 *de*	0.00 *f*	0.08 *de*	0.02 *f*
SA3780	0.11 *abc*	0.02 *f*	0.12 *abc*	0.02 *f*	0.16 *abc*	0.02 *f*

Supplementary Table 10. Net effect of combined Verticillium infection with V31-2 strain and temperature at infection, on weight of pods per plant (g) for seven *M. truncatula* lines. 20°C, 25°C and 28°C are the temperatures of infection and first four weeks of growth. C : control condition, I : inoculated. Different letters indicate a significant difference at P<0,05.

	20°C		25°C		28°C	
Line	C	I	C	I	C	I
A17	10 *abc*	7 *bcd*	6 *abc*	5 *bcd*	11 *abc*	10 *bcd*
PRT180-A	5 *def*	4 *def*	3 *def*	4 *def*	7 *def*	7 *def*
DZA45.5	8 *abc*	5 *abc*	7 *abc*	10 *abc*	13 *abc*	10 *abc*
SA9048	4 *cde*	2 *ef*	5 *cde*	1 *ef*	7 *cde*	7 *ef*
F83005.5	12 *a*	2 *ef*	12 *a*	2 *ef*	15 *a*	5 *ef*
DZA315.16	9 *ab*	4 *ef*	9 *ab*	0.3 *ef*	16 *ab*	2 *ef*
SA3780	11 *a*	0.4 *f*	10 *a*	0.3 *f*	15 *a*	0.8 *f*

Supplementary Table 11. Net effect of combined Verticillium infection with V31-2 strain and temperature at infection, on dry aerial biomass per plant (g) for seven *M. truncatula* lines. 20°C, 25°C and 28°C are the temperatures of infection and first four weeks of growth. C : control condition, I : inoculated. Different letters indicate a significant difference at P<0,05.
